# Activation of Cilia-Independent Hedgehog/GLI1 Signaling as a Novel Concept for Neuroblastoma Therapy

**DOI:** 10.3390/cancers13081908

**Published:** 2021-04-15

**Authors:** Anke Koeniger, Anna Brichkina, Iris Nee, Lukas Dempwolff, Anna Hupfer, Ilya Galperin, Florian Finkernagel, Andrea Nist, Thorsten Stiewe, Till Adhikary, Wibke Diederich, Matthias Lauth

**Affiliations:** 1 Center for Tumor- and Immune Biology, Department of Gastroenterology, Philipps University Marburg, 35043 Marburg, Germany; anke.koeniger@imt.uni-marburg.de (A.K.); anna.brichkina@staff.uni-marburg.de (A.B.); anna.hupfer@gmx.de (A.H.); ilya.galperin@imt.uni-marburg.de (I.G.); 2 Department of Medicinal Chemistry and Center for Tumor- and Immune Biology, Philipps University Marburg, 35043 Marburg, Germany; nee.i@gmx.de (I.N.); dempwolff@pm.me (L.D.); wibke.diederich@staff.uni-marburg.de (W.D.); 3 Center for Tumor- and Immune Biology, Bioinformatics Core Facility, Philipps University Marburg, 35043 Marburg, Germany; finkernagel@imt.uni-marburg.de; 4 Member of the German Center for Lung Research (DZL), Center for Tumor- and Immune Biology, Genomics Core Facility, Institute of Molecular Oncology, Philipps University Marburg, 35043 Marburg, Germany; andrea.nist@imt.uni-marburg.de (A.N.); stiewe@uni-marburg.de (T.S.); 5 Institute for Biomedical Informatics and Biostatistics, Philipps University Marburg, 35043 Marburg, Germany; adhikary@imt.uni-marburg.de; 6 Core Facility Medicinal Chemistry, Philipps University Marburg, 35043 Marburg, Germany

**Keywords:** neuroblastoma, hedgehog, GLI1, GLI3, primary cilia, ISX, ISX9, HDAC

## Abstract

**Simple Summary:**

Elevated *GLI1* expression levels are associated with improved survival in NB patients and *GLI1* overexpression exerts tumor-suppressive traits in cultured NB cells. However, NB cells are protected from increased GLI1 levels as they have lost the ability to form primary cilia and transduce Hedgehog signals. This study identifies an isoxazole (ISX) molecule with primary cilia-independent GLI1-activating properties, which blocks NB cell growth. Mechanistically, ISX combines the removal of GLI3 repressor and the inhibition of class I HDACs, providing proof-of-principle evidence that small molecule-mediated activation of GLI1 could be harnessed therapeutically in the future.

**Abstract:**

Although being rare in absolute numbers, neuroblastoma (NB) represents the most frequent solid tumor in infants and young children. Therapy options and prognosis are comparably good for NB patients except for the high risk stage 4 class. Particularly in adolescent patients with certain genetic alterations, 5-year survival rates can drop below 30%, necessitating the development of novel therapy approaches. The developmentally important Hedgehog (Hh) pathway is involved in neural crest differentiation, the cell type being causal in the etiology of NB. However, and in contrast to its function in some other cancer types, Hedgehog signaling and its transcription factor GLI1 exert tumor-suppressive functions in NB, rendering GLI1 an interesting new candidate for anti-NB therapy. Unfortunately, the therapeutic concept of pharmacological Hh/GLI1 pathway activation is difficult to implement as NB cells have lost primary cilia, essential organelles for Hh perception and activation. In order to bypass this bottleneck, we have identified a GLI1-activating small molecule which stimulates endogenous GLI1 production without the need for upstream Hh pathway elements such as Smoothened or primary cilia. This isoxazole compound potently abrogates NB cell proliferation and might serve as a starting point for the development of a novel class of NB-suppressive molecules.

## 1. Introduction

Neuroblastoma (NB), a pediatric cancer affecting the peripheral nervous system, is the most common type of malignancy to be diagnosed in the first year of life [1,2]. NB originates from embryonic neural crest cells failing to terminally differentiate along the sympathoadrenal lineage, giving rise to an expanding pool of premature neuroblasts. This process is triggered by several genetic events such as *MYCN* gene amplification or activating *ALK* mutations, among others [3,4,5,6]. Clinically, NB presents as a surprisingly heterogeneous disease classified in distinct stages with vastly differing outcomes. While NB stages 1 and 4s have a low risk and a 5-year survival of more than 90%, stage 4 is associated with a high risk and a poor survival of patients, particularly if they are older or harbor *MYCN* amplifications [7]. As such, animals ectopically expressing *MYCN* or mutant *ALK* represent common models of NB [8]. Despite novel treatment modalities which also include targeted immune therapy against the oncofetal antigen GD2, curing high risk NB still represents a clinical challenge and novel and innovative treatment ideas are needed to improve long-term outcome in the future.

The Hedgehog (Hh) signaling pathway is essential for correct embryonic development, tissue patterning and organ formation. In the canonical signaling cascade, Hh ligands (SHH, DHH, IHH) bind to Patched (PTCH1 and PTCH2) receptors, which are localized to a solitary, microtubule-held membrane protrusion, the primary cilium [9,10]. Binding of ligands to PTCH1 activates Smoothened (SMO), a step presumably involving cholesterol transport across the ciliary membrane [11]. Active, ciliary-localized SMO transmits the signal via changes in cAMP levels to the latent transcription factors GLI2 and GLI3, which induce target gene transcription. An important target gene is represented by *GLI1*, which aids in the amplification of the Hh transcriptional output and might also possess its own target genes [12]. In the central nervous system (CNS), Hh signaling often promotes neural specification, as best evidenced in the patterning of the neural tube, where Hh exerts ventralizing activity defining the identity of prospective motor neurons [13]. In the peripheral nervous system (PNS), several neural crest cell populations (e.g., trunk and cephalic ones) are SHH-responsive and Hh also contributes to differentiation and specification steps [14,15]. As such, Hh was proposed to exert pro-differentiating and tumor-suppressive functions in NB [16,17,18,19], although other reports suggested an oncogenic role of Hh/GLI in NB [20,21,22,23,24].

Here, we demonstrate that higher *GLI1* expression is associated with a good prognosis in NB patients and that ectopic expression of GLI1 abrogates NB cell growth, making small molecule GLI1 activators potentially attractive for future applications in NB therapy and other settings in which elevated Hh signaling is beneficial, such as in regenerative medicine. In recent years however, and due to the oncogenic role of unrestrained Hh signaling in malignancies such as medulloblastoma or basal cell carcinoma, the development of small molecule Hh pathway inhibitors has widely surpassed the identification of compounds able to activate Hh signaling [25,26]. In the meantime, preclinical and clinical Hh antagonists have been discovered, which block the signaling process along the entire cascade. In contrast, Hh agonists are restricted to the modulation of upstream pathway elements such as SMO or PTCH1 [27]. For instance, the agonistic compounds SAG [28] or Purmorphamine [29] both target the heptahelical transmembrane region of SMO [30], making the presence of a primary cilium mandatory for drug function. As such, these agents are inactive in cilia-less cells (such as those in NB) or are less effective on cell populations with low cilia frequencies.

Following this concept, we identified an isoxazole molecule (ISX) which is capable of inducing GLI1 expression in cilia-less human and mouse cells and which functions through the combined abrogation of GLI3 repressor and HDAC class I functionality, resulting in pronounced growth-inhibitory effects in NB cells. The characterization of this drug and its associated working mechanism could serve as a starting point for the future development of therapeutic compounds acting as downstream Hh/GLI agonists for the application in regenerative medicine and in selected malignancies.

## 2. Materials and Methods

### 2.1. Cell Lines

NIH3T3, AsPC1 were purchased from CLS and IMR32, SH-SY5Y, MCF7, 22Rv1, DU145 cells were purchased from ATCC. MEF cells were kindly provided by Wade Bushman [31]. All cell lines were cultured in Dulbecco’s Modified Eagle Medium (DMEM (high glucose plus glutamine and pyruvate), Invitrogen (Carlsbad, CA, USA)) supplemented with 10% fetal bovine serum (FBS) and 1% penicillin/streptomycin at 37 °C with 5% CO_2_. If not otherwise stated, serum concentrations were reduced to 0.5% during experiments for all cell types. All cells were regularly checked for mycoplasma contamination.

### 2.2. Reagents

Inhibitors were purchased from the following companies: From Biomol (Hamburg, Germany): SAG; MS-275; CI-994; Chidamide; MC-1568; TMP-195; Bufexamac. From Sigma: SAHA (Vorinostat); SANT1; Purmorphamine; 20- and 25-Hydroxycholesterol; Nicotinamide. From R&D Biosystems: recombinant SHH (C24II). From Cayman Chemicals (Ann Arbor, MI, USA): Epigenetics Screening Library (96-Well); TSA (Trichostatin); JQ1. From AppliChem: Apicidin; Solvent (DMSO). ISX was synthesized in-house by Wibke Diederich.

### 2.3. Generation of Knockout Cell Lines via CRISPR/Cas9

For generating *Kif3a* knockout NIH3T3 cells the CRISPR/Cas9 gene editing system was used (target sequence underlined): Primers mKif3a_Crispr1_s (5′-CACCGAAGCTGCGATAATGTGAAGG) and mKif3a_Crispr1_as (5′-AAACCCTTCACATTATCGCAGCTTC) were annealed and cloned into the BbsI site of pU6-(BbsI)-CBh-Cas9-T2A-mCherry vector (Addgene: #64324). The KO cells were generated according to a strategy described in [32]. To this end, NIH3T3 cells were transfected with the cloned plasmid (pU6-mKif3a_Crispr1), pTia-2A-Hygro (kindly provided by Till Adhikary) and empty pU6-(BbsI)-CBh-Cas9-T2A-mCherry (ratio 1:1:0.5) using Helix-In transfection reagent (Oz Biosciences, OZB-HX10500) for 24 h. Subsequently, cells were selected with Hygromycin (VWR International GmbH, J60681.MC) at a concentration of 400 µg/mL until resistant clones appeared. Single cell clones were picked and the knockout was confirmed via Western blot, immunofluorescence and in functional RT-qPCR. *GLI1*-deficient SH-SY5Y cells were generated accordingly using a CRISPR construct targeting the sequence ATCCCACATCCTCAGTCCCG plus a pTia-CMV-Blast construct.

### 2.4. Colony Assays

Transfection of neuroblastoma cells was done using Helix-IN (Biozol) according to the manufacturer’s protocol. SH-SY5Y cell were transfected with EF-GLI1, EF-GLI1^ZFmut^ (zinc finger mutant) or EF-MCS (empty vector control). The cells were subsequently selected with Blasticidin (Capricorn Scientific GmbH (Ebsdorfergrund, Germany)) at a concentration of 5 µg/mL to enrich for transfected cells. Approx. 4–5 d after transfection/treatment cells were fixed with 4% formaldehyde/PBS at RT for 10 min, followed by a PBS wash. Then, 10% Giemsa solution was added and cell were stained for 15 min at RT, followed by several washing steps with water to remove excess staining solution. Afterwards, culture plates were air-dried.

### 2.5. Hh/GLI Reporter Assays

MCF7 cells were transfected with 8xGLI-BS-Luc or 8xmutGLI-BS-Luc (mutated GLI-binding sites) using TransIT-2020 (Mirus Bio (Madison, WI, USA)) according to the manufacturer’s protocol. One day later, cells were treated with 20 µM ISX in 10% FBS-containing medium for 24 h. Subsequently, cell were lysed in Passive Lysis Buffer (Promega (Madison, WI, USA)) and firefly activity was measured using an Orion L microplate luminometer (Berthold Detection Systems) using Beetle-Juice Luciferase Assay (PJK Biotech (Kleinblittersdorf, Germany)). For normalization the total protein concentration was measured in a Bradford assay (Bio-Rad (Hercules, CA, USA)) with a bovine serum albumin (BSA) standard curve.

### 2.6. Epigenetic Library Screening

MCF7 cells were transfected with 8xGLI1-BS-Luc plasmid on larger wells as described above. Then, 2 days later, 15,000 cells/96-well were plated in white 96-well plates with clear bottom. Then, cells were treated in 5% FBS-containing medium for 24 h with 10 µM of an (extended) epigenetic drug library (Cayman, #11076) (final DMSO conc. 0.5%). Subsequently, cells were lysed in Passive Lysis Buffer (Promega) and firefly luciferase was determined on a plate-reading luminometer using BeetleJuice reagent (PJK Biotech). For normalization, an aliquot of the lysate was used for total protein determination (Bradford assay, Bio-Rad).

### 2.7. RNA/cDNA Analysis

Total RNA was extracted using the NucleoSpin RNA II kit from Macherey-Nagel according to the manufacturer’s protocol. 1 µg of total RNA was used for cDNA synthesis using the iScript cDNA Synthesis Kit (BioRad). For quantitative PCR reactions the Absolute QPCR SYBR Green Mix (Thermo scientific (Waltham, MA, USA)) was used. qPCR reactions were performed on 96 well plates using either the Mx3000P or Mx3005P qPCR systems (Agilent (Santa Clara, CA, USA)). Relative expression was calculated according to the 2^ΔΔCt^- method.

### 2.8. Western Blotting

Separation of lysates by SDS-PAGE (Bio-Rad) and subsequent blotting on Immobilon-PVDF membranes (Millipore) was done as described in [33], followed by incubation with the respective primary antibody. The following primary antibodies were used: GLI1 (Cell Signaling Technology (CST), # 2534), GLI2 (R&D Systems, #AF3635-S), GLI3 (R&D Systems (Minneapolis, MN, USA), #AF3690), SUFU (CST; #2522), Acetyl-Histone H3 (Lys27) (CST, # 8173), total Histone H3 (Active Motif (Carlsbad, CA, USA), #ACM-39052), KIF3A (CST, #8507), β-Actin (Sigma, #A5441) and α-Tubulin (Sigma, #T6199). After incubation with a corresponding horseradish peroxidase (HRP)-coupled secondary antibody (CST, # #7076 and #7074; Santa Cruz, # sc-2350) the HRP signal was detected using Pierce ECL Western Blotting Substrate (Thermo Scientific) according to the manufacturer’s protocol.

### 2.9. Small Interfering RNA (siRNA) Transfections

Cells were transfected with 35 nM siRNA (purchased from Dharmacon (Lafayette, CO, USA) or Sigma (St. Louis, MO, USA)) using RNAiMax (Invitrogen (Carsbad, CA, USA)). Control siRNA was from Qiagen (Hilden, Germany) (All-Stars siRNA; siCon). For siRNA sequences, please refer to the Supplemental Materials Table S1.

### 2.10. Microscopy

Cells were seeded on etched cover slips and treated according to the respective experimental protocol. Subsequently, cells were fixed in 4% formaldehyde/PBS (10 min at room temperature (RT), washed, permeabilized with 0.5% Triton-X100/PBS at RT for 5 min, and blocked with 10% serum/PBS for 1 h at RT. Then, cover slips were incubated with the primary antibody (Acetylated tubulin (AcTub); Sigma #T6793) in antibody-solution (PBS containing 10% serum and 0.1% Saponin) overnight at 4 °C. After washing with PBS at RT, cover slips were incubated with fluorophore-coupled secondary antibodies diluted in antibody-solution at RT in the dark for 2 h. After washing with PBS and rinsing with water, cover slips were mounted with mounting medium containing 4′,6-Dia (midin-2-phenylindol (DAPI) (Vectashield). Microscopy was performed on a Leica DM 5500 Wide field microscope (Leica Microsystems, Wetzlar, Germany) using 3D deconvolution. Resulting z-stacks are presented as maximum intensity projections.

### 2.11. HDAC Assay

For analysis of whole cells, 2 × 10^4^ cells/well were seeded in white 96-well plates with clear bottom and treated with ISX, SAHA or DMSO in 5% FBS-containing medium. Histone deacetylase (HDAC) activity was determined using the HDAC-Glo I/II Assay-Kit (Promega) according to the manufacturer’s protocol. For normalization of total protein amount, identical parallel wells were measured using a Bradford assay (Bio-Rad).

HDAC activity in nuclear lysates was determined after isolation of nuclei from non-treated cells according to [34] and nuclear lysis in 1%Triton-X100/PBS.

### 2.12. HAT Assay

Following the respective treatment, MCF7 subcellular cell fractions were prepared using a protocol from [34]. At the end, nuclei were lysed in PBS/1% Triton-X100 and the protein concentration was measured in a Bradford assay using a BSA standard curve. For analysis of HAT activity, the Histone Acetyltransferase (HAT) Assay Kit (Sigma, EPI001) was used according to the manufacturer’s protocol.

### 2.13. Analysis of Histone Modifications (ModSpec)

IMR32 cells were cultured in 0.5% FBS-containing growth medium and treated with DMSO or 20 µM ISX for 8 h. Subsequently, cells were trypsinized, spun down, and the cell pellet was shipped to Active Motif for contract histone post-translational modifications (PTM) quantitation service.

### 2.14. Neuroblastoma Tissue Staining

Formalin-fixed and paraffin-embedded tissue were sectioned, de-paraffinized and antigens were retrieved using boiling 10 mM citrate (pH 6). Subsequently, sections were permeabilized with 0.2% Triton X100/PBS (5 min, RT) and blocked with 10% serum/PBS (1 h, RT). Primary antibody staining was overnight (4 °C) using anti-acetylated α-tubulin (Sigma T6793) and anti-pericentriolar material 1 (PCM1; CST #5213) antibodies, followed by a 1–2 h incubation at RT with fluorescently labelled secondary antibodies (Invitrogen). Sections were embedded in Vectashield/DAPI and visualized on a Leica DM 5500 Wide field microscope (Leica Microsystems, Wetzlar, Germany) using 3D deconvolution. Resulting z-stacks are presented as maximum intensity projections. The use of human patient material through the Marburg Biobank was approved by the local ethics committee at the University Hospital Marburg.

### 2.15. EdU Staining

Cell proliferation (EdU incorporation) was determined using the Click-IT EdU Alexa Fluor 488 Imaging kit (Life Technologies (Carlsbad, CA, USA)) according to the manual.

### 2.16. RNA Sequencing

Integrity of total RNA was assessed on the Bio-Rad Experion. Sequencing libraries were prepared with the TruSeq Stranded mRNA Kit (Illumina (San Diego, CA, USA)). On-board cluster generation using the TruSeq Rapid SR Cluster Kit-HS (Illumina) and single read 50 nucleotide sequencing was performed on a HiSeq Rapid SR Flow Cell (Illumina) on the Illumina 1500 platform. Human genome assembly (hg38) and gene model data were retrieved from Ensembl release 81 (MCF7) or 86 (IMR32). RNAseq data were aligned using STAR 2.4.1a [35]. Gene expression was quantified within the exonic regions of protein coding genes in terms of counts per million (CPM).

### 2.17. Medicinal Chemistry

Information on compound synthesis is provided in the Supplementary Materials Section.

### 2.18. cAMP Determination

Levels of cyclic AMP (cAMP) were determined with the cAMP-GLO assay kit from Promega according to the instructions given by the manufacturer.

### 2.19. Statistics and Data Accessibility

Statistical comparisons were made of *n* ≥ 3 experiments using an unpaired two-tailed Student’s *t*-test (MS Excel or GraphPad Prism). Significances were indicated as ns (not significant; *p* > 0.05), * *p* < 0.05, ** *p* < 0.01, *** *p* < 0.001. Kaplan–Meier curves and gene expression analyses/gene correlations from public datasets were done using the R2: Genomics Analysis and Visualization Platform (http://r2.amc.nl; accessed on 1 January 2021).

RNAseq data sets are accessible at ArrayExpress: MCF7 (±ISX): E-MTAB-10248; IMR32 (±ISX): E-MTAB-10249.

## 3. Results

### 3.1. GLI1 Expression Suppresses NB Growth

**Figure 1:**
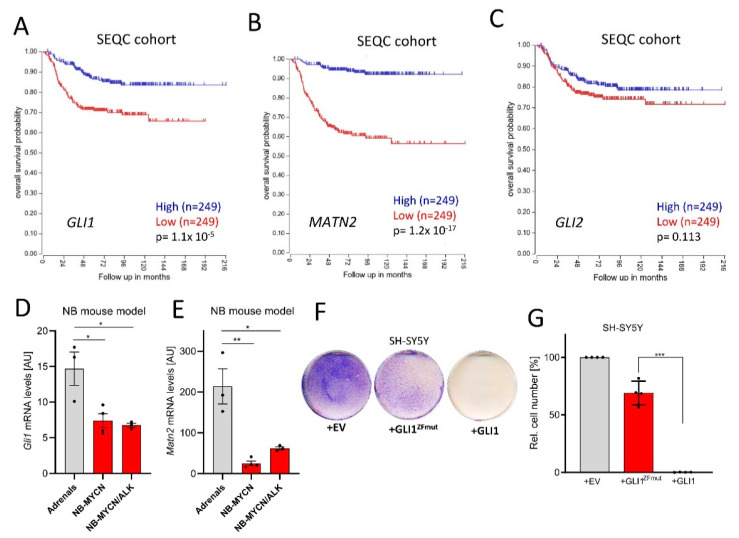
GLI1 exerts tumor-suppressive roles in neuroblastoma (NB). (**A**) Kaplan–Meier curves depicting overall survival of NB patients in relationship to the *GLI1* expression (SEQC cohort, median split, logrank test). (**B**) Kaplan–Meier curves depicting overall survival of NB patients in relationship to the *MATN2* expression (SEQC cohort, median split, logrank test). (**C**) Kaplan–Meier curves depicting overall survival of NB patients in relationship to the *GLI2* expression (SEQC cohort, median split, logrank test). (**D**) *Gli1* mRNA expression in normal mouse adrenals, *Mycn*-induced murine NB or *Mycn/ALK*-induced murine NB (Heukamp et al., 2012). Shown is the mean ± SD of 3–4 animals. (**E**) *Matn2* mRNA expression in normal mouse adrenals, *Mycn*-induced murine NB or *Mycn/ALK*-induced murine NB (Heukamp et al., 2012). Shown is the mean ± SD of 3–4 animals. (**F**) Colony stain (blue) of SH-SY5Y cells transfected with empty control vector (EV), GLI1^ZFmut^ (DNA binding-deficient) or wildtype *GLI1*. One representative experiment of *n* = 4 is depicted. (**G**) Quantification of the experiment shown in (**F**). Mean of *n* = 4 ± SD. Significances were indicated as * *p* < 0.05, ** *p* < 0.01, *** *p* < 0.001.

In order to clarify whether Hh/GLI activity exerts pro- or anti-tumorigenic roles in NB, we investigated a publicly available dataset of 498 NB patients (SEQC cohort; GSE49711; GSE62564 [36]). In this dataset, high *GLI1* expression was clearly and significantly associated with a better overall survival (Figure 1A). Similar findings were also made for *MATN2* (Figure 1B), a GLI1 target gene in NB ([16] and Figure S1A for gene correlation). However, the expression of *GLI2* did surprisingly not significantly associate with NB patient survival, possibly explaining some of the uncertainties around Hh/GLI and NB in the field. Using a second independent NB dataset including 476 patients [37], the above findings were identically validated (Figure S2A–C). In line with earlier findings [17], the positive association of *GLI1* levels with survival was selective for patients without *MYCN* gene amplification (Figure S2D,E). Nevertheless, we further found that compared to normal adrenal tissue, the levels of *Gli1* and its target gene *Matn2* were downregulated in two mouse models of NB [38] (Figure 1D,E), despite the involvement of *Mycn* overexpression. Finally, we overexpressed human *GLI1* in human SH-SY5Y NB cells (Figure 1F). As a control, we used either empty vector or a GLI1 construct carrying mutations in its zinc-finger domain (GLI1^ZFmut^), rendering this variant functionally inactive as a transcription factor since it is unable to bind to DNA. As can be seen in Figure 1F,G, only the transfection of transcriptionally competent GLI1 led to a block in NB proliferation, despite comparable expression levels of the two constructs (Figure S2F). Taken together, these data strongly support a transcription-dependent tumor-suppressive role for GLI1 in the more frequent NB cases not harboring *MYCN* amplifications.

### 3.2. NB Cells Lack Primary Cilia and Are Not Hh-Responsive

**Figure 2:**
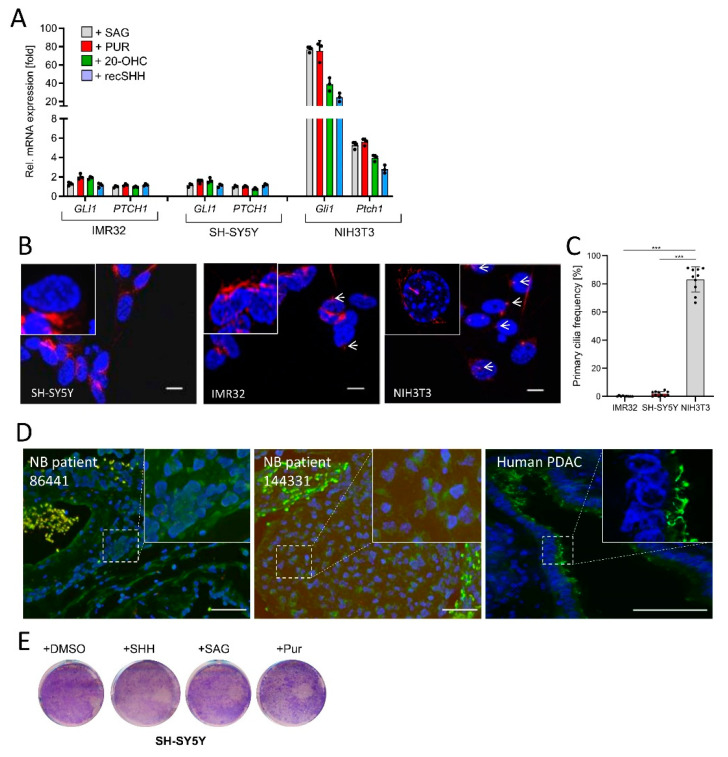
NB cells are Hh-unresponsive and lack primary cilia. (**A**) Hh target gene expression (*GLI1, PTCH1*) in IMR32 and SH-SY5Y cells exposed to SAG (100 nM), Purmorphamine (PUR, 2 µM), 20(S)-Hydroxy-Cholesterol (20-OHC, 10 µM) or recombinant SHH (recSHH, 0.4 µg/mL), each for 48 h. Shown is the fold change in mRNA expression compared to solvent. Mouse NIH3T3 cells were used as positive control. Values are mean of *n* = 3 ± SD. (**B**) Immunofluorescent images of IMR32, SH-SY5Y and NIH3T3 cells starved for 48 h in 0.5% FBS-containing medium and stained for the primary cilium marker acetylated α-Tubulin (AcTub, in red). Nuclei appear in blue (DAPI). Scale bar 10 µm. White arrows depict primary cilia. (**C**) Quantification of primary cilia as shown in (**B**). At least *n* = 300 cells of each cell line were counted. Each dot represents a field of view. Mean ±SD. (**D**) Primary cilia (AcTub, green)/Basal body (PCM1, red) staining of human NB tissue. Human pancreatic cancer tissue was used as positive control (AcTub, green). Nuclei appear in blue (DAPI). Scale bar in each panel is 50 µm. (**E**) Colony stain of SH-SY5Y cells treated with DMSO, SAG (100 nM), Purmorphamine (PUR, 2 µM), or recombinant SHH (recSHH, 0.4 µg/mL) for 5d. One example of two is shown. Significances were indicated as *** *p* < 0.001.

Based on the findings above, therapeutic stimulation of Hh/GLI signaling might appear as an attractive novel approach in NB treatment. Therefore, we tested the impact of four different Hh agonists (SAG, Purmorphamine, 20-Hydroxycholesterol, recombinant SHH) acting on either PTCH1 or SMO. However, none of these agonists was capable of eliciting a Hh signaling response in IMR32 or SH-SY5Y human NB cells (as measured by the expression of Hh target genes (*GLI1, PTCH1*)), despite the fact that all of these compounds were fully functional in NIH3T3 fibroblasts, a cell line which we used as positive control (Figure 2A). As many tumor entities display a loss of primary cilia on their cancer cells [39,40,41], we investigated the frequency of primary cilia in the two human NB cell lines (Figure 2B). In line with their lack of Hh-responsiveness, there was a drastic drop in primary cilia frequencies in IMR32 and SH-SY5Y cells when compared to NIH3T3 cells, which displayed a cilia frequency of more than 80% (Figure 2C). This lack of cilia occurrence was also observed in two human NB tissue samples, where human pancreatic cancer tissue was used as a positive control and where primary cilia were readily detectable in the ductal epithelium (Figure 2D). These data implied that NB cells lost their ability to form primary cilia and were therefore unable to receive signals from agonists acting at the level of PTCH1 or SMO. As an expected consequence of these findings, treating NB cells with the panel of four different Hh agonists had no effect on their overall proliferation (Figure 2E).

### 3.3. Identification of a Novel Downstream GLI1 Activating Compound

**Figure 3:**
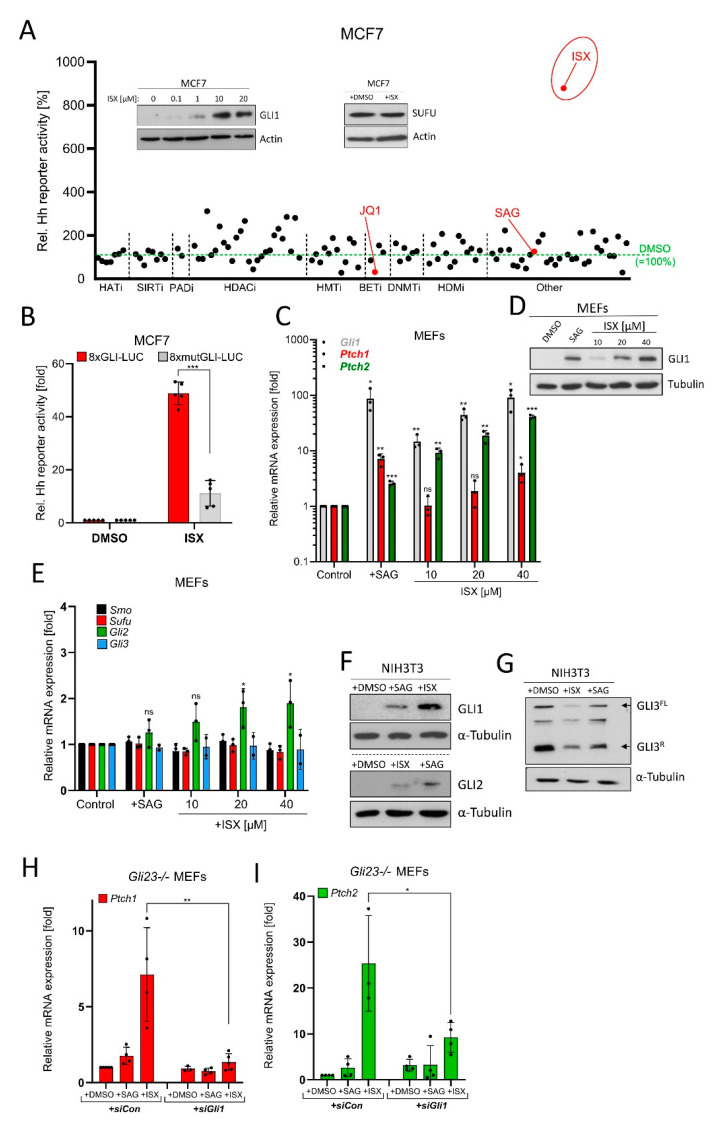
Identification of a small molecule GLI1 inducer. (**A**) Epigenetic drug screen in MCF7 cells transiently transfected with 8xGLI-Luc reporter plasmid and treated with compounds for 24 h. Shown are mean values of *n* = 3 relative to DMSO treatment (=100%). HATi: Histone acetyl transferase inhibitors; SIRTi: Sirtuin inhibitors; PADi: Protein arginine deiminase inhibitors; HDACi: Histone deacetylase inhibitors; HMTi: Histone methyl transferase inhibitors; BETi: BET-domain inhibitors; DNMTi: DNA methyl transferase inhibitors; HDMi: Histone demethylase inhibitors. See Supplementary Materials Section for more details. The insets depict Western blots of MCF7 cell lysates after treatment with DMSO or ISX (48 h) as indicated. In the SUFU blot experiment and in the screen, ISX was used at a concentration of 20 µM. (**B**) Luminometric Hh reporter assay in MCF7 transiently transfected with 8xGLI-Luc or mutated 8xGLI-Luc construct. Cells were treated with DMSO or 20 µM ISX for 24 h. Shown is the mean of *n* = 5 ± SD. (**C**) Hh target gene expression (*Gli1, Ptch1, Ptch2*) in mouse embryonic fibroblasts (MEFs) treated for 48 h as measured by qPCR. SAG: 100 nM. Mean of *n* = 3 ± SD. (**D**) GLI1 Western blot of MEF lysates. Treatment as indicated; SAG: 100 nM. One representative experiment of two. (**E**) Hh pathway gene expression (*Smo, Sufu, Gli2, Gli3*) in mouse embryonic fibroblasts (MEFs) treated for 48 h as measured by qPCR. SAG: 100 nM. Mean of *n* = 3 ± SD. (**F**) Western blot depicting GLI1 and GLI2 protein levels in treated (ISX 20 µM; SAG 100 nM; 48 h) NIH3T3 cells. Shown is one representative experiment of *n* = 3. (**G**) Western blot depicting GLI3 protein levels in treated (ISX 20 µM; SAG 100 nM; 48 h) NIH3T3 cells. GLI3^FL^: Full-length GLI3; GLI3^R^: Repressor form of GLI3. Shown is one representative experiment of *n* = 3. (**H**) Quantitative PCR measuring expression of the Hh target gene *Ptch1* in *Gli23-/-* MEFs. Treatment with ISX (20 µM) or SAG (100 nM) for 48 h. Cells were transfected with control siRNA (siCon) or an equimolar pool of three *Gli1*-specific siRNAs (siGli1). Mean of *n* = 4 ± SD. (**I**) Quantitative PCR measuring expression of the Hh target gene *Ptch2* in *Gli23-/-* MEFs. Treatment with ISX (20 µM) or SAG (100 nM) for 48 h. Cells were transfected with control siRNA (siCon) or an equimolar pool of three *Gli1*-specific siRNA (siGli1). Mean of *n* = 4 ± SD. Significances were indicated as ns (not significant; *p* > 0.05), * *p* < 0.05, ** *p* < 0.01, *** *p* < 0.001.

Our experiments so far implied that a small molecule compound which could bypass the primary cilium and still stimulate GLI1 could be of interest in the setting of NB. To this end, we envisioned that the chromatin might serve as a reasonable target for such a molecule as it is functionally acting downstream of primary cilia. Therefore, we screened an extended epigenetic drug library for its impact on a luminometric Hh reporter construct carrying GLI binding sites (GLI-BSs). Here, we used human MCF7 breast carcinoma cells as a tool as they are readily transfectable, do not possess primary cilia (see later), and are Hh-unresponsive (Figure S3A,B). The drug screen revealed known downstream inhibitors of Hh/GLI activity such as JQ1 [42] and proved the inefficacy of the SMO agonist SAG on cilia-less cells, validating the conditions of the screen (Figure 3A). Most importantly, we identified a small isoxazole molecule (ISX, a.k.a. ISX9; Figure S3C), which has previously been described as a proneural compound [43]. This substance induced the Hh reporter much more potently than all other compounds and dose-dependently induced the expression of GLI1 protein (Figure 3A, inset). Importantly, this effect was not mediated by a drug-mediated loss of Suppressor-of-Fused (SUFU), a crucial downstream antagonist of Hh signaling [44] (Figure 3A, inset). Validating these findings in follow-up experiments revealed that ISX also did induce a GLI-BS-defective reporter construct (8xmutGLI-Luc), but at much lower strengths than the construct containing functional GLI binding sites (8xGli-Luc; Figure 3B). We went on to characterize this small molecule in additional cell systems and could observe strong ISX-induced endogenous Hh target gene stimulation (*Gli1, Ptch1, Ptch2*) (Figure 3C) and GLI1 protein expression (Figure 3D) in mouse embryonic fibroblasts (MEFs). Importantly, ISX did not nonspecifically upregulate all Hh pathway members as the expression of e.g., *Smo*, *Sufu* or *Gli3* was unaltered (Figure 3E). Next, we analyzed the impact of ISX on GLI protein levels and GLI3 processing in NIH3T3 cells, a widely used cellular model in the Hh field [45,46,47,48]. As can be seen in Figure 3F, ISX strongly induced GLI1 and only very modestly GLI2. Strikingly, it also reduced the levels of GLI3 repressor (GLI3^R^) but apparently did not stabilize full-length GLI3 (GLI3^FL^), as does for instance the SMO agonist SAG (Figure 3G). In turn, overexpression of GLI3^R^ reduced the ability of ISX to induce a Hh reporter in NIH3T3 cells (Figure S3D). Quantification of the GLI3 effects suggested that ISX somehow destabilized the entire GLI3 pool and did not impact on GLI3 processing (Figure S3E). In line with this hypothesis, ISX did not alter intracellular cAMP levels (Figure S3F).

Finally, we wished to more directly examine whether the ISX-induced GLI1 protein was functionally active. To this end, we made use of *Gli2*/*Gli3* double knockout MEFs [31] measuring the induction of the GLI target genes *Ptch1* and *Ptch2*. As expected, ISX was able to induce *Ptch1/2* gene expression in these cells whereas SAG was inactive (Figure 3H,I). However, the additional transfection with a pool of three different *Gli1*-specific siRNAs blocked the effect of ISX on *Ptch1/2* expression, indicating that ISX required GLI1 activity for its impact on *Ptch1/2*. This in turn demonstrated that ISX indeed induced a transcriptionally competent form of GLI1 in these cells (Figure 3H,I). In conclusion we identified ISX, a small molecule compound capable of inducing functional GLI1 in human and mouse cells.

### 3.4. ISX Functions Independently of Primary Cilia and Smoothened

**Figure 4:**
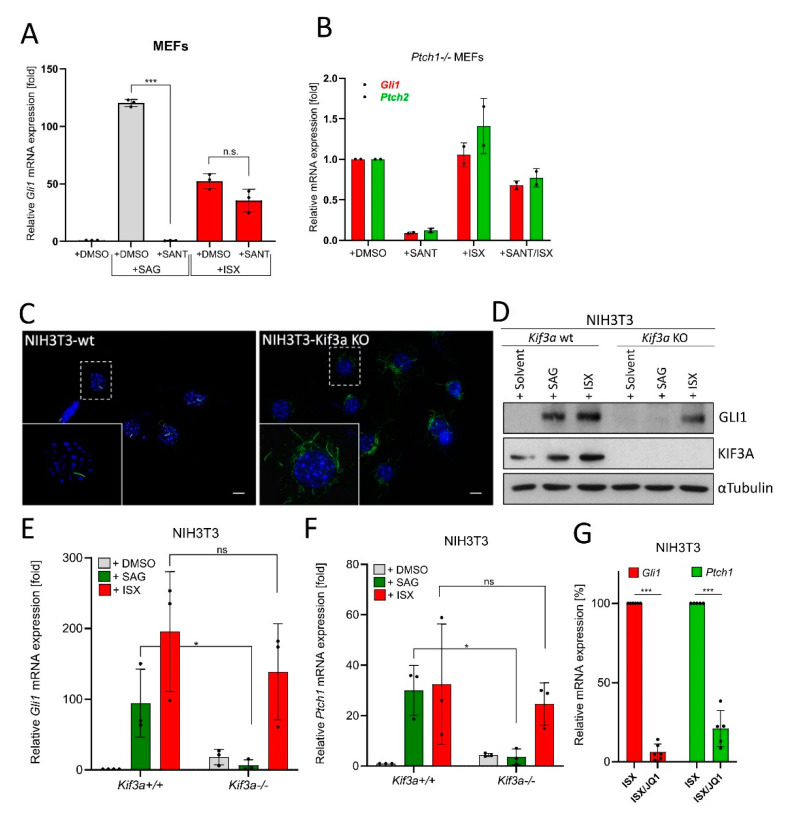
ISX functions independently of primary cilia. (**A**) Expression of the Hh target gene *Gli1* in MEFs as measured by qPCR. Cells were treated with ISX (20 µM), SANT1 (0.2 µM) or SAG (100 nM) for 48 h. Shown is the mean of *n* = 3 ± SD. (**B**) Expression of the Hh target gene *Gli1 and Ptch2* in *Ptch1-/-* MEFs as measured by qPCR. Cells were treated with ISX (20 µM), SANT1 (0.2 µM) or SAG (100 nM) for 48 h. Shown is the mean of *n* = 2 ± SD. (**C**) Primary cilia (AcTub, green) in wildtype (wt) and *Kif3a* knockout (KO) NIH3T3 cells. Nuclei appear in blue (DAPI). Scale bar is 10 µm. (**D**) Immunoblot depicting the protein levels of GLI1 and KIF3A in *Kif3a* wt/KO NIH3T3 cells. Treatment was for 48 h with 100 nM SAG or 20 µM ISX. Shown is one of three independent experiments. Tubulin was used for normalization. (**E**) Expression of *Gli1* in *Kif3a* wt/KO NIH3T3 cells. Treatment as in (**D**). Mean of *n* = 3 ± SD. (**F**) Expression of *Ptch1* in *Kif3a* wt/KO NIH3T3 cells. Treatment as in (**D**). Mean of *n* = 3 ± SD. (**G**) Expression of *Gli1 and Ptch1* in NIH3T3 cells treated with ISX (20 µM) or ISX (20 µM)/JQ1 (1 µM) for 48 h. The ISX-induction was set to 100%. Mean of *n* ≥ 5 ± SD. Significances were indicated as ns (not significant; *p* > 0.05), * *p* < 0.05, *** *p* < 0.001.

In a next step, we specifically queried the cilia-independence of ISX function. To this end, MEF cells were treated with the SMO antagonist SANT1 [28] to block Hh signaling at the level of primary cilia. As can be seen in Figure 4A (and Figure S4A), while the effects of the SMO agonist SAG were fully abrogated under SANT1 treatment (as expected), ISX was still capable of inducing Hh signaling in the presence of SMO inhibition. Similar findings were made when we used MEF cells with constitutive SMO activation due to the genetic depletion of *Ptch1* [49] (Figure 4B). We continued our studies by generating NIH3T3 cells lacking *Kif3a*, a crucial component in cilia formation [50] (Figure 4C). In agreement with our previous data, ISX was capable of inducing GLI1 protein expression in wildtype and *Kif3a* knockout (KO) cells while SAG selectively lost this ability in KO cells (Figure 4D). This result was also verified on the Hh target gene level (*Gli1, Ptch1*) (Figure 4E,F) and could additionally be verified in transient *Kif3a*-knockdown experiments (Figure S4B,C). Finally, as upstream inhibition of Hh signaling by SANT1 or loss of cilia could not block ISX effects, we tested the BET-domain inhibitor JQ1, which functions at the level of *GLI1/2* transcription [42]. In line with the results from our drug screen (Figure 3A), JQ1 was capable of blocking ISX function and abrogated ISX-induced Hh target gene stimulation (Figure 4G).

### 3.5. ISX Functions on a Wide Range of Different Human Cell Types

**Figure 5:**
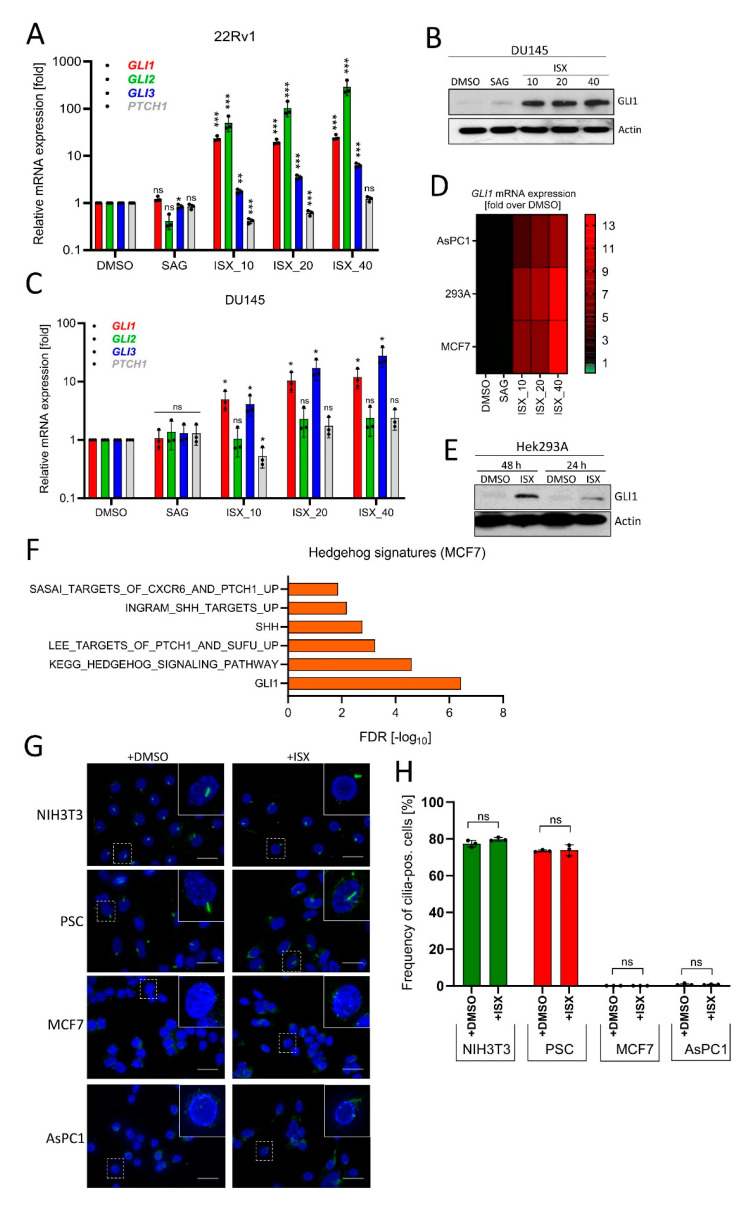
ISX activates GLI1 in a wide range of cell types. (**A**) Expression of Hh pathway genes in human prostate carcinoma cells (22Rv1). Treatment for 48 h with the indicated concentration of ISX (i.e., ISX_10: 10 µM ISX). SAG: 100 nM. Mean of *n* = 3 ± SD. (**B**) GLI1 immunoblot of treated DU145 cells. ISX concentrations (numbers) in [µM]. SAG: 100 nM. (**C**) Expression of Hh pathway genes in human prostate carcinoma cells (DU145). Treatment for 48 h with the indicated concentration of ISX (i.e., ISX_10: 10 µM ISX). SAG: 100 nM. Mean of *n* = 3 ± SD. (**D**) Heatmap summary of *GLI1* expression (qPCR) in pancreatic AsPC1, breast MCF7 and kidney Hek293A cells. ISX concentrations [µM] as in (A). SAG: 100 nM. Shown is the mean of *n* = 3 (AsPC1; 293A) and *n* = 2 (MCF7) experiments. (**E**) GLI1 protein immunoblot of Hek293A cells treated for the indicated times. ISX: 20 µM. Shown is one representative example of two. (**F**) Hh-related transcriptome signatures (RNAseq) of ISX (20 µM, 48 h)-treated MCF7 cells versus DMSO control. (**G**) Primary cilia (AcTub, green) in various cell lines. Treatment with 20 µM ISX for 48 h. Nuclei appear in blue (DAPI). Scale bar is 10 µm. (**H**) Quantification of primary cilia as seen in (**G**). Shown is the mean of *n* = 3 experiments with at least 100 cells being counted for each condition in each experiment. Significances were indicated as ns (not significant; *p* > 0.05), * *p* < 0.05, ** *p* < 0.01, *** *p* < 0.001.

In order to investigate how broad the action of ISX really is with respect to different cell types, we tested its effects on a panel of human cell lines. We found that ISX readily induced GLI1 protein as well as Hh target genes in the human prostate carcinoma cell lines 22Rv1 and DU145 (Figure 5A–C). Dose-dependent *GLI1* mRNA induction was also observed in human pancreatic cancer cells (AsPC1), in human breast carcinoma cells (MCF7), and in human embryonic kidney cells (Hek293A) (Figure 5D). GLI1 protein induction in the latter cells was already detectable after 24 h of ISX treatment and increased further when treatment was extended to 48 h (Figure 5E). Furthermore, we asked whether Hh signatures could be observed in a more global approach such as transcriptomic profiling by means of RNAseq. As illustrated in Figure 5F, when using MCF7 cells as an example we could clearly detect significant ISX-induced upregulation of general Hh-related terms such as *‘GLI1’*, or ‘KEGG_Hedgehog Signaling Pathway’, underscoring the role of ISX as an inducer of endogenous Hh signaling. Eventually, we wanted to rule out the possibility that ISX functions as a ‘re-ciliating’ compound capable of inducing de novo cilia growth in cells. Therefore, we analyzed two human cilia-less cell lines (MCF7 and AsPC1) plus two ciliated cell lines (murine NIH3T3 cells and human pancreatic stellate cells (PSC)) (Figure 5G). In these experiments, ISX had no impact on the cilia frequency of any cell line investigated (Figure 5H), strongly arguing against a general cilium-inducing activity of this compound.

In order to address the chemical space of the ISX compound, we performed a series of structural modifications that allowed us to deduce structure-activity relationships (SAR). To this end, we modified the thiophene moiety, the cyclopropyl moiety and replaced the central core element with a number of different heteroaromatic ring systems (Figure S5A). Replacement of the thiophene with a methyl group (Ox-01) or other alkyl substituents (Ox-38) resulted in a complete loss of activity, while bio-isosteric replacement with a phenyl moiety (Ox-02) was tolerated. Remarkably, all further modifications to this phenyl substituent reduced the activity again (Ox-25, Ox-26, Ox-29, Ox-31, Ox-32, Ox-36, Ox-37). Replacing the cyclopropyl moiety with smaller or slightly larger alkyl substituents (Ox-03, Ox-04, Ox-06, Ox-10, Ox-11, Ox-14–Ox-18, Ox-22, Ox-27) also had a huge impact on activity. Of all the synthesized compounds within this series, only the *N*-ethyl substituted derivative (Ox-22) showed activity, albeit reduced. Modification of the central core element (Ox-20, Ox-21, Ox-24, Ox-26, Ox-28) or amide functionality (Ox-05, Ox-07–Ox-09) was also not tolerated. Unfortunately, as described, none of the 29 synthesized analogues showed equal or even improved biological activity in a cellular assay compared to ISX (Figure S5B). Most of the compounds showed a drastic loss of activity, even in cases where only subtle structural changes were made. These results indicate that the binding mode of the ISX compound is very complex or stringent and does not allow for structural flexibility.

In summary, we could show that ISX functions in a broad range of cilia-less cell types to induce GLI1 expression, that it did not affect ciliogenesis and that medicinal chemistry efforts to improve this compound are challenging.

### 3.6. ISX Possesses HDAC Inhibitory Potential

**Figure 6:**
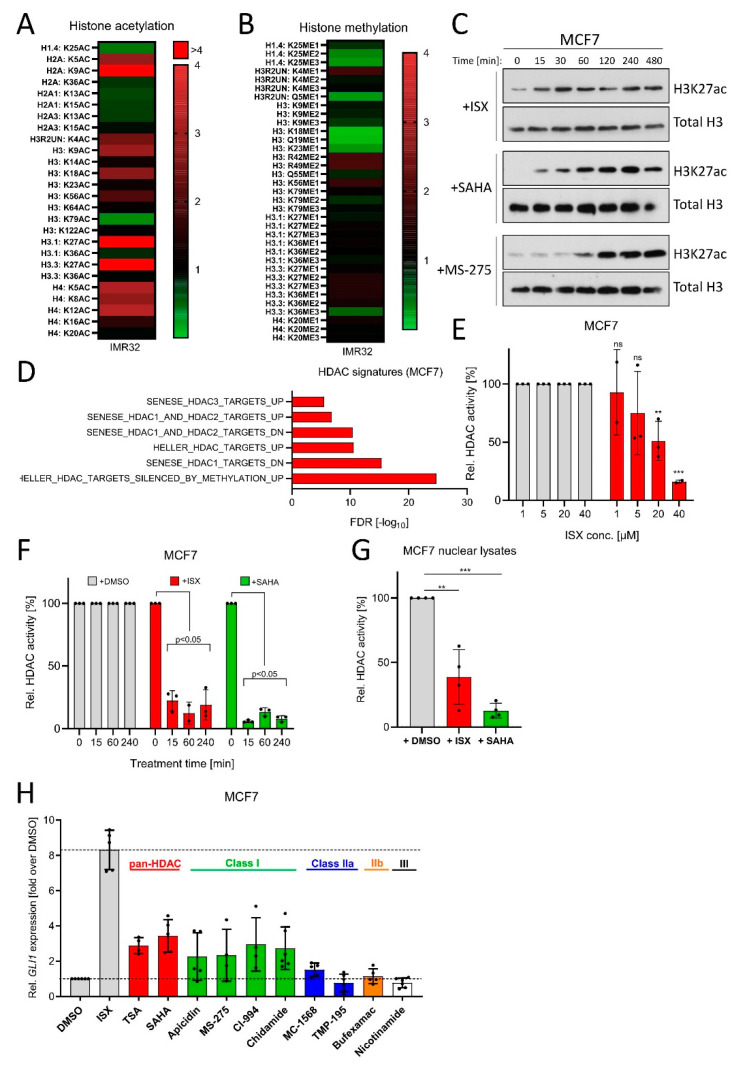
ISX possesses HDAC inhibitory functions. (**A**) Mass spectrometric determination of acetylated histone residues in IMR32 cells. Treatment with ISX (20 µM) for 8 h. (**B**) Mass spectrometric determination of methylated histone residues in IMR32 cells. Treatment with ISX (20 µM) for 8 h. (**C**) Western blot analysis of H3K27 acetylation kinetics in MCF7 cells treated with ISX (20 µM), SAHA (1 µM) or MS-275 (1 µM). Total histone H3 levels were used as normalizing control. Shown is one representative of *n* = 3 experiments. (**D**) HDAC-related transcriptome signatures (RNAseq) of ISX (20 µM, 48 h)-treated MCF7 cells versus DMSO control. (**E**) Cellular HDAC class I/II activity in MCF7 cells exposed to the indicated concentrations of ISX for 1 h. Shown is one experiment measured in duplicate/triplicate (mean ± SD). DMSO controls were set to 100%. (**F**) Cellular HDAC class I/II activity in MCF7 cells exposed to 20 µM ISX (or 5 µM SAHA) for the indicated times. Shown is one experiment measured in duplicate/triplicate (mean ± SD). DMSO controls were set to 100%. (**G**) Class I/II HDAC activity in isolated and lysed MCF7 nuclei, which were treated in vitro (10 min, RT) with DMSO (set to 100%), 20 µM ISX or 5 µM SAHA. Shown is one of *n* = 2 experiments measured in quadruplicate (mean ± SD). (**H**) *GLI1* expression in MCF7 cells treated for 24 h with the indicated compounds. ISX: 20 µM; TSA: 0.5 µM; SAHA: 0.5 µM; Apicidin: 0.25 µM; MS-275: 1 µM; CI-994: 10 µM; Chidamide: 5 µM; MC-1568: 5 µM; TMP-195: 1 µM; Bufexamac: 50 µM; Nicotinamide: 10 mM. Mean of at least *n* = 4 ± SD. Significances were indicated as ns (not significant; *p* > 0.05), ** *p* < 0.01, *** *p* < 0.001.

Next, we went on to shed more light on the molecular mechanism of ISX. Based on the functional experiments performed before, we assumed a function at the level of transcriptional regulation, at least to some extent. Therefore, we performed an unbiased mass spectrometric-based screen of histone modifications induced by ISX. As can be seen in Figure 6A, ISX led to a strong upregulation of histone acetylation, not limited to but particularly pronounced at the residues H3K27 and H2AK9. Histone methylation was instead not strongly induced (Figure 6B). The induction of H3K27 acetylation (H3K27ac) (which is typically associated with transcriptional activation) was confirmed by Western blotting in MCF7 cells (Figure 6C). Intriguingly, the kinetics of H3K27ac accumulation was different than the reference compounds (the pan-HDAC inhibitor SAHA and the class I-specific inhibitor MS-275) and was much faster (Figure 6C). An ISX-mediated induction of HDAC-specific terms (e.g., ‘HELLER_HDAC targets silenced by methylation_UP’) was also observed in MCF7 transcriptome datasets (Figure 6D). Since all these experiments pointed towards a possible HDAC-inhibitory function of ISX, we performed HDAC class I/II-activity assays on intact MCF7 cells. Indeed, ISX dose- (Figure 6E) and time-dependently (Figure 6F) blocked HDAC activity in these cells already after 15 min of treatment, suggesting a direct effect. We therefore repeated the experiment with isolated and detergent-solubilized nuclear fractions and could also observe a significant inhibition of HDAC activity upon 10 min of ISX in vitro addition (Figure 6G). In order to compare the GLI1-effects of HDAC inhibition with those of ISX, we treated MCF7 cells with a range of different HDAC inhibitors (Figure 6H). While inhibition of the HDAC classes IIa, IIb and III had no significant impact on *GLI1* induction, inhibition of class I or pan-HDAC inhibition resulted in stimulation of *GLI1* transcription, albeit much weaker than that obtained with ISX (Figure 6H). Because a recent report claimed that ISX functions as a histone acetyl transferase (HAT) activator rather than an HDAC inhibitor [51], we tested the cellular HAT activity in MCF7 cells (Figure S6A). Indeed, ISX enhanced nuclear HAT activity, but this effect did not reach significance and the same trend was also observed for the pan-HDAC inhibitor SAHA, suggesting that the increase in HAT activity might be the compensatory result of HDAC inhibition [52,53]. In summary, we concluded that ISX induced histone acetylation through its class I HDAC inhibitory function. While the inhibition of HDACs likely contributed to the *GLI1*-inducing effects of ISX, other aspects were additionally required to achieve full induction.

### 3.7. ISX Induces GLI1 in NB Cells and Blocks Cell Growth

**Figure 7:**
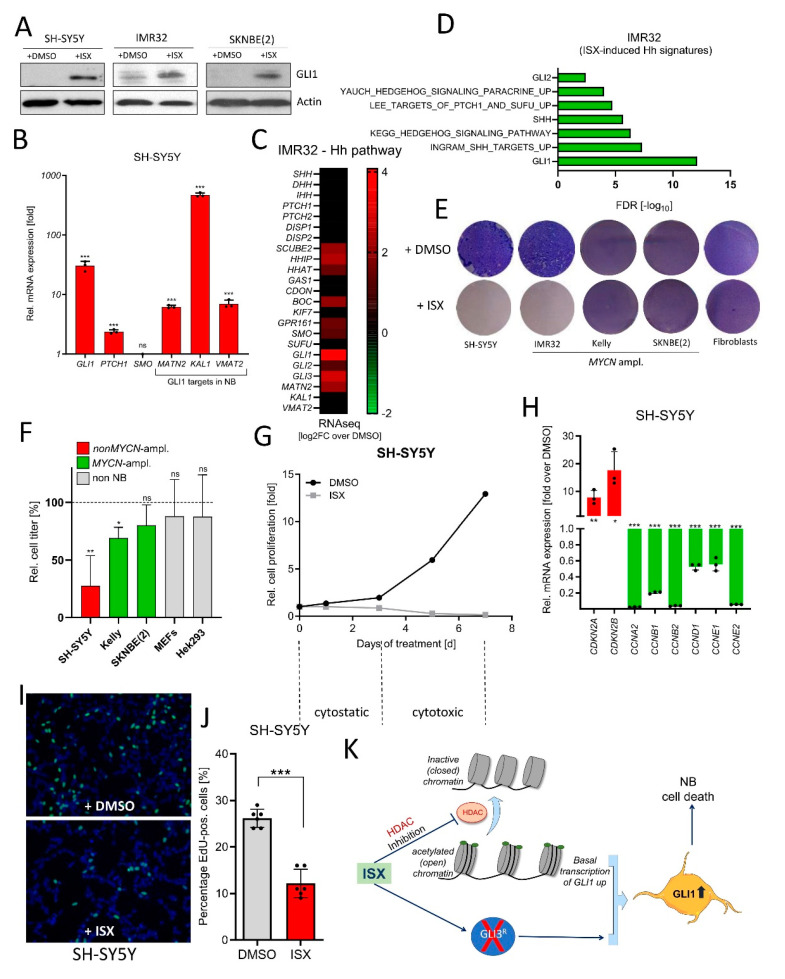
ISX blocks NB cell proliferation. (**A**) Western blot depicting GLI1 protein levels in two different human NB cell lines after ISX (20 µM, 48 h) treatment. Shown are the representative results of one experiment of two. (**B**) Expression of Hh pathway genes (qPCR) in ISX (20 µM, 48 h)-treated SH-SY5Y cells. Mean of *n* = 3 ± SD. (**C**) Expression (log2 fold change over DMSO) of Hh pathway genes (RNAseq) in ISX (20 µM, 48 h)-treated IMR32 cells. (**D**) Hh-related transcriptome signatures (RNAseq) of ISX (20 µM, 48 h)-treated IMR32 cells versus DMSO control. (**E**) Colony stain (Giemsa, blue) of the indicated cell lines exposed to DMSO or ISX (20 µM) for 4–5 d. Fibroblasts were human pancreatic stellate cells (PSCs). Shown is one representative experiment of two. (**F**) Relative cell count changes in SH-SY5Y cells upon DMSO or ISX (20 µM) treatment. Shown is the mean of *n* = 2 experiments. (**G**) Relative cell count changes in IMR32 cells upon DMSO or ISX (20 µM) treatment. Shown is the mean of *n* = 2 experiments. (**H**) Changes in cell cycle gene expression (qPCR) in ISX (20 µM, 48 h)-treated SH-SY5Y cells. Mean of *n* = 3 ± SD. (**I**) BrDU (EdU) labelling (green) of DMSO/ISX-treated SH-SY5Y cells. Nuclei appear in blue (DAPI). ISX concentration was 20 µM; treatment time 24 h (last 4 h with 10 µM EdU). (**J**) Quantification of EdU labelling experiment depicted in (**I**). Shown are two counted regions with at least 100 cells of each of *n* = 3 independent experiments (±SD). (**K**) General scheme on the mechanism of ISX function. ISX inhibits class I HDACs and removes GLI3 repressor, two events which might synergize to drive GLI1 expression. Significances were indicated as ns (not significant; *p* > 0.05), * *p* < 0.05, ** *p* < 0.01, *** *p* < 0.001.

Finally, we wanted to test the functional impact of ISX treatment on NB cells. In agreement with our previous data, ISX induced GLI1 protein (Figure 7A) and Hh pathway genes (Figure 7B) in several human NB cells. In addition, transcriptome analysis by means of RNAseq revealed the ISX-stimulated upregulation of Hh pathway genes (mostly strongly of *GLI1*) (Figure 7C) and the induction of Hh-dependent transcriptome signatures (Figure 7D) in IMR32 cells. In contrast to upstream Hh pathway agonists (Figure 2E), ISX was also able to strongly reduce NB cell proliferation of the non *MYCN*-amplified SH-SY5Y cell line (Figure 7E). This was not the case in two out of three *MYCN*-amplified cell lines (SKNBE(2), Kelly). Non-cancerous control cell lines such as fibroblasts or Hek293 cells were not grossly affected in their growth by ISX treatment (Figure 7E,F). In order to assess the contribution of *GLI1* induction to the overall growth inhibitory effects of ISX, we generated *GLI1*-deficient SH-SY5Y cells (Figure S7A). Indeed, *GLI1*-KO cells were much less sensitive to ISX than *GLI1*-WT cells, albeit some anti-proliferative effect of ISX remained (Figure S7B). ISX-induced cell growth inhibition in SH-SY5Y cells was biphasic: in the first 2–3 days, ISX caused a cytostatic effect whereas a cytotoxic period followed afterwards during the days 3–7 (Figure 7G). Transcriptionally, ISX reduced the expression of many *Cyclin* genes such as *CCNA2*, *CCNB2* or *CCNE2* and induced the expression of the negative cell cycle regulators *CDKN2A* and *CDKN2B* (Figure 7H). Moreover, EdU-labelling revealed a significant reduction in NB cell proliferation upon ISX treatment (Figure 7I,J). Taken together, ISX induced GLI1 in NB cells and could strongly interfere with NB cell proliferation.

## 4. Discussion

The Hedgehog (Hh) pathway is an embryonic signaling cascade regulating many developmentally crucial steps, mostly tissue patterning and cell differentiation [12]. In the adult organism, Hh activity is restricted to sites of tissue repair and stem cell niches. Hence, activating Hh has proven beneficial in the regenerative settings of cardiac and limb ischemia [54,55], skin wound healing [56,57] as well as tendon and bone fracture repair [58,59]. Furthermore, Hh expands the stem cell pools needed for subsequent repair processes, as shown after spine injury [60] or in the retina [61]. Moreover, active Hh signaling is involved in regeneration after lung injury and in the maintenance of physiological pulmonary tissue quiescence [62]. In addition, Hh signaling is suppressed in Down Syndrome patients and pharmacological induction alleviates major symptoms in mouse models, suggesting novel areas of potential Hh agonist treatments [33,63,64,65].

On the other side, overactivated Hh signaling is associated with the development of basal cell carcinoma and medulloblastoma [66,67], but evidence for a tumor-restraining activity of (mostly stromal) Hh signaling has also been provided for pancreatic [68,69,70], colon [71] and pulmonary [72,73,74] malignancies. Another example where Hh exerts tumor-suppressive effects is neuroblastoma (NB), the most frequent solid cancer in infants and young children [16,18]. Indeed, we could observe an in vitro NB cell growth repression upon expression of GLI1. A DNA binding-defective GLI1 mutant was much less capable to do so (although some small effect could be seen, which might reflect residual activity, Figure 1G). Our observation of GLI1 as a tumor suppressor is in agreement with other reports on Hh signaling in NB (Souzaki et al., 2010; Gershon et al., 2009; Oue et al., 2010; Paul et al., 2013). Additional studies on this topic often used pharmacological compounds to block GLI function (Wickström et al., 2013; Mao et al., 2009; Schiapparelli et al., 2011; Xu et al., 2012), making the interpretation of specificity issues difficult. Furthermore, future work is needed to clarify the specific role of GLI2 in NB, which might be different than the one which GLI1 fulfills in this disease. In general, pharmacological tools to induce GLI1 seem beneficial not only in regenerative but even in certain cancerous settings. Noteworthy, with respect to NB the tumor-suppressive effects of GLI activation might be restricted to the large subset of patients not harboring *MYCN* gene amplifications (Souzaki et al., 2010).

However, from a drug development standpoint the vast majority of small molecule Hh modulators function as antagonists of the pathway, a result of the widespread interest for Hh/GLI in connection with its role in tumorigenesis [25,26]. Currently, a therapeutic induction of Hh/GLI would only be possible by application of recombinant Hh ligands or the small molecules SAG [28], Purmorphamine [29], certain glucocorticoids [75] or oxysterols [76,77]. All of these molecules have in common that they bind to the upstream pathway elements PTCH1 or SMO and therefore require the presence of a functional primary cilium. This in turn makes Hh activation in cancer cells challenging as primary cilia are often lost, including NB, necessitating the use of Hh/GLI activators capable to bypass the primary cilium.

Here, we report on the identification of a small isoxazole (ISX) molecule which is capable of inducing *GLI1* gene expression in the absence of primary cilia and independent of PTCH1 or SMO. According to our knowledge, a compound with such properties has not been described before. Mechanistically, ISX promotes the loss of the GLI3 repressor form (GLI3^R^), which by itself would already stimulate *GLI1* expression. In addition, ISX inhibits HDAC activity (presumably of class I HDACs), thereby creating an open chromatin and favoring *GLI1* transcription, which might synergize with the removal of GLI3^R^ (Figure 7K). At this point we cannot rule out that further mechanisms also contribute to the overall effects of ISX as well. In addition, the HDAC-inhibitory nature of ISX necessarily brings along certain Hh-unrelated effects, and future experiments might allow to formulate more specific GLI1 agonists. Structure-activity relationship (SAR) studies however revealed that the binding mode of ISX might be complex and/or might potentially involve more than one drug target. Further optimization of this compound might also allow to design a GLI1-inducing agent acting selectively on the PNS while not penetrating the blood-brain barrier. HDACs have previously been reported as positive modulators of GLI function by directly affecting GLI acetylation [78,79,80]. We currently speculate that ISX might be able to overcome this negative regulation by inducing counteracting measures or by simply generating sufficient GLI1 protein which might be less active but still suffices for transcriptional activation [80,81].

Interestingly, the ISX compound identified in this study has previously been reported as a neurogenesis-promoting substance [43,82,83,84,85]. Moreover, class I HDAC inhibition (which would also increase *GLI1* expression) induces NB differentiation [86]. In light of the fact that Hh signaling is involved in neuron specification it will be interesting to learn to what degree the GLI1-inducing capability of ISX contributes to its general proneural impact. In summary, we describe for the first time a primary cilium-independent small molecule GLI1 inducer which functions in a wide range of human and mouse cell types.

## Data Availability

The data presented in this study are openly available at ArrayExpress: MCF7 (±ISX): E-MTAB-10248; IMR32 (±ISX): E-MTAB-10249.

## Supplementary Materials

The following are available online at https://www.mdpi.com/article/10.3390/cancers13081908/s1, Figure S1: *MATN2* as a GLI1 target gene. Figure S2: *GLI1*-related survival in NB. Figure S3: ISX effects on GLI3. Figure S4: ISX and primary cilia. Figure S5: Structure-activity relationship (SAR) of ISX. Figure S6: ISX and HAT activity. Figure S7: GLI1-dependency of ISX. Table S1: RLU values from epigenetic drug screen.
