# Supplementary material for: Activation of Cilia-Independent Hedgehog/GLI1 Signaling as a Novel Concept for Neuroblastoma Therapy
Source: Cancers (Basel). 2021 Apr 15;13(8):1908. doi: 10.3390/cancers13081908 (PMC8071409; doi:10.3390/cancers13081908)
Supplement: Supplementary file 1 [file cancers-13-01908-s001.zip › cancers-1161632-supplementary.pdf]

Koeniger et al.

Supplemental figure legends

Figure S1:

(A) Correlation between *GLI1* and *MATN2* expression in NB tumors (SEQC cohort, 498 patients).

Figure S2:

(A) Kaplan Meier curves depicting overall survival of NB patients in relationship to the *GLI1* expression (Kocak cohort [37], median split, logrank test).

(B) Kaplan Meier curves depicting overall survival of NB patients in relationship to the *MATN2* expression (Kocak cohort [37], median split, log rank test).

(C) Kaplan Meier curves depicting overall survival of NB patients in relationship to the *GLI2* expression (Kocak cohort [37], median split, log rank test).

(D) Kaplan Meier curves depicting overall survival of NB patients harboring *MYCN* amplifications in relationship to the *GLI1* expression (SEQC cohort, median split, log rank test).

(E) Kaplan Meier curves depicting overall survival of NB patients not harboring *MYCN* amplifications in relationship to the *GLI1* expression (SEQC cohort, median split, log rank test).

(F) Quantification of *GLI1* expression in transfected SH-SY5Y cells (qPCR). Related to Fig. 1F,G. Shown is the mean ±SD of n=3-4 experiments.

Figure S3:

(A) Hh target gene expression (*GLI1, PTCH1*) in MCF7 cells treated with SAG (100 nM), Purmorphamine (PUR; 2 µM), 25-Hydroxy-cholesterol (25-OHC; 5 µM) or recombinant SHH (recSHH; 0.4 µg/ml) for 48 h. Mean of n=3 ±SD.

(B) *Gli1* mRNA expression in NIH3T3 cells treated for 48 h with the indicated compounds to verify their effectiveness. SAG (100 nM), Purmorphamine (PUR; 2 µM), 25-Hydroxy-cholesterol (25-OHC; 5 µM), recombinant SHH (recSHH; 0.4 µg/ml). Shown is one experiment.

(C) Chemical structure of ISX.

(D) Luminometric Hh activity assay (8xGLI-Luc) in transiently transfected NIH3T3 cells. Cells were treated with 20 µM ISX for 24 h. Shown is the mean of n=3 ±SD. Significance by paired 2-tailed t-test.

(E) Quantification of $GLI3^{R}/GLI3^{FL}$ protein levels in treated NIH3T3 cells. Shown is the mean ±SD of n=2-4 experiments. ISX: 20 µM; SAG: 100 nM. Treatment time: 48h.

(F) Quantification of cAMP concentration in NIH3T3 cells. Equal cell numbers were treated for 1h with the indicated compounds. ISX_20/40 = 20/40 µM ISX. Controls: FSK=Forskolin (Adenylate Cyclase activator, 50 µM), SQ22536 (Adenylate Cyclase inhibitor, 100 µM). Shown is one experiment of two measured in quadruplicate.

Figure S4:

(A) Expression of the Hh target gene *Ptch1* in MEFs as measured by qPCR. Cells were treated with ISX (20 µM), SANT1 (0.2 µM) or SAG (100 nM) for 48 h. Shown is the mean of n≥3 ±SD.

(B) Expression of *Gli1* in MEFs as measured by qPCR. Cells were transfected with either control siRNA (siCon) or with a pool of four *Kif3a*-specific siRNAs (siKif3a) followed by treatment with DMSO, ISX (20 µM), or SAG (100 nM) for 48h. Shown is the mean of n=3 ±SD.

(C) Measurement of *Kif3a*-knockdown efficiency in MEFs. Mean of n=3 ±SD.

Figure S5:

(A) Chemical structures of ISX-analogs.

(B) Heatmap representation of the biological activities of ISX and its analogs (20 µM, 48 h treatment time) as measured by *GLI2* induction in human PaTu8988T pancreatic cancer cells. The ISX-induced values were set to 100%. Shown is the mean of a duplicate measurement of one experiment of 2-4 independent experiments.

Figure S6:

(A) HAT assay of nuclear extracts derived from MCF7 cells treated with DMSO, ISX (20 µM) or SAHA (1 µM) for 1 h. Shown is one experiment measured in quadruplicate (mean ±SD).

Figure S7:

(A) GLI1 immunoblot of *GLI1* wildtype (WT) and knockout (KO) SH-SY5Y cells. Shown is one experiment of two. ISX treatment (20 µM) was for 48h.

(B) Cell titer determination of *GLI1* WT/KO SHSY5Y cells exposed to the indicated concentrations of ISX for 4 d (5% FBS). Shown is one representative measurement in triplicate of n=2 experiments.

A

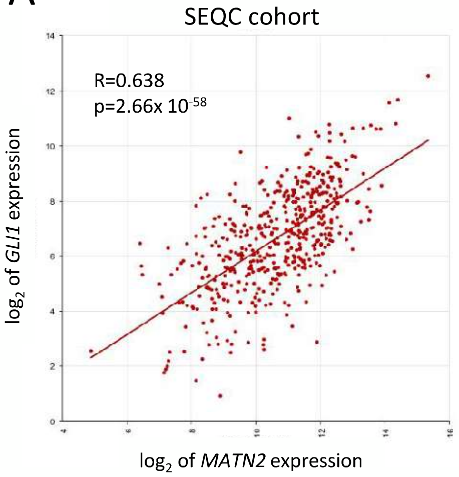
SEQC cohort
R=0.638
p=2.66x 10-58
log2 of GLI1 expression
log2 of MATN2 expression
14
12
10
8
6
4
2
0
4
6
8
10
12
14
16

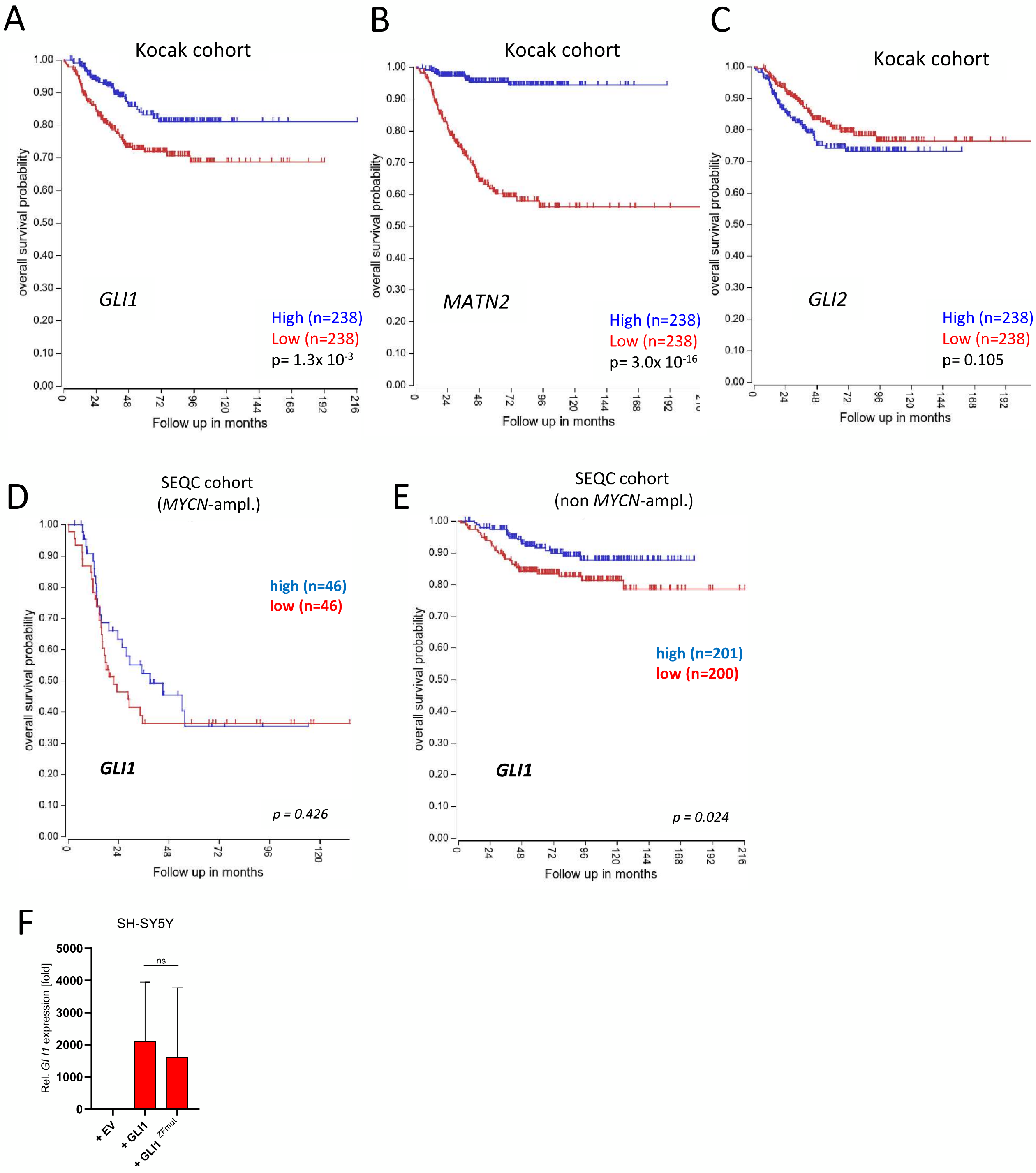
A
Kocak cohort
overall survival probability
GLI1
High (n=238)
Low (n=238)
p= 1.3x 10-3
Follow up in months
B
Kocak cohort
overall survival probability
MATN2
High (n=238)
Low (n=238)
p= 3.0x 10-16
Follow up in months
C
Kocak cohort
overall survival probability
GLI2
High (n=238)
Low (n=238)
p= 0.105
Follow up in months
D
SEQC cohort
(MYCN-ampl.)
high (n=46)
low (n=46)
overall survival probability
GLI1
p = 0.426
Follow up in months
E
SEQC cohort
(non MYCN-ampl.)
overall survival probability
high (n=201)
low (n=200)
GLI1
p = 0.024
Follow up in months
F
SH-SY5Y
Rel. GLI1 expression [fold]
ns
+ EV
+ GLI1
+ GLI1 ZFmut

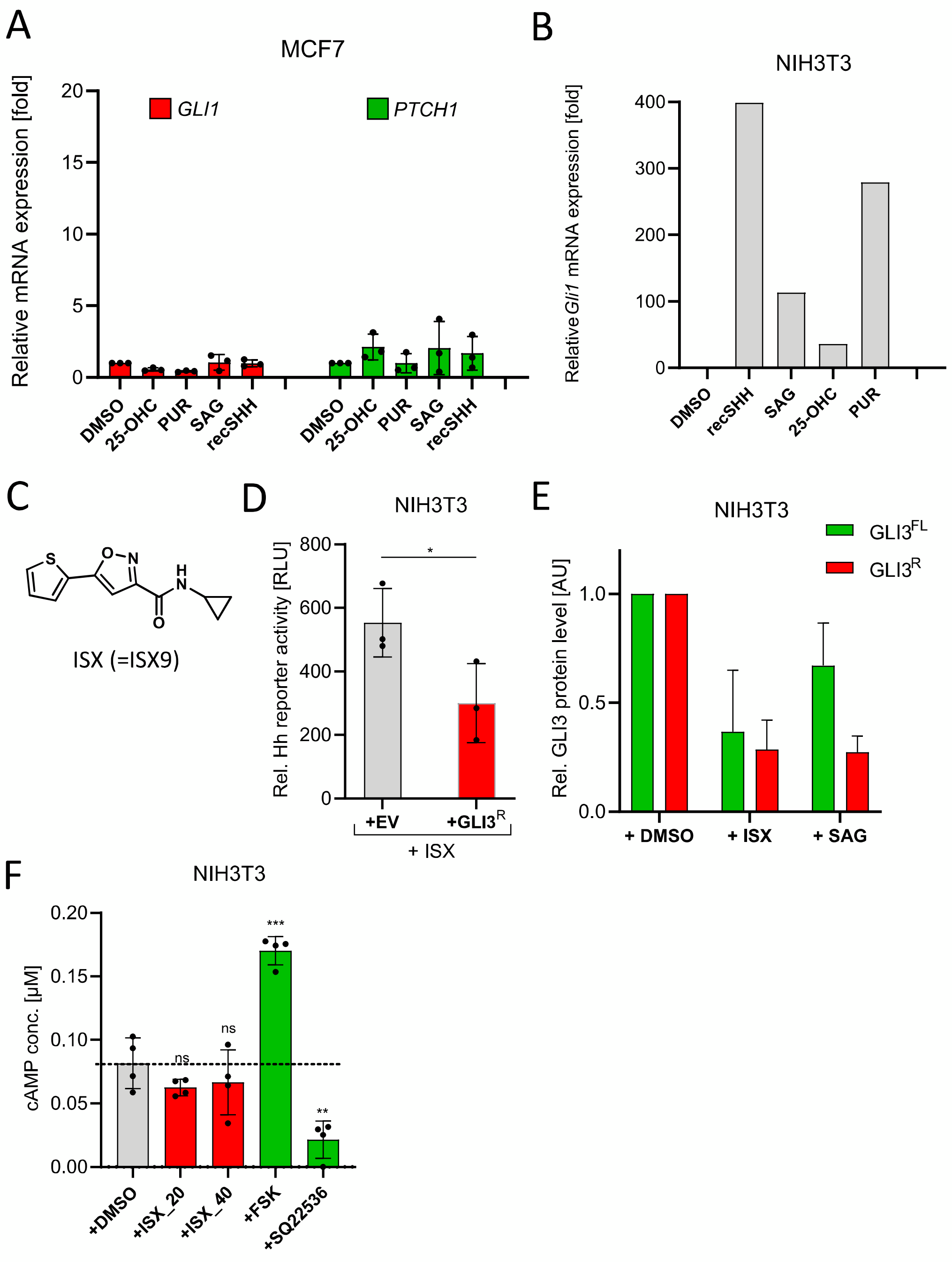
A
MCF7
Relative mRNA expression [fold]
GLI1
PTCH1
DMSO
25-OHC
PUR
SAG
recSHH
B
NIH3T3
Relative Gli1 mRNA expression [fold]
DMSO
recSHH
SAG
25-OHC
PUR
C
ISX (=ISX9)
D
NIH3T3
Rel. Hh reporter activity [RLU]
+EV
+GLI3R
+ ISX
E
NIH3T3
GLI3FL
GLI3R
Rel. GLI3 protein level [AU]
+ DMSO
+ ISX
+ SAG
F
NIH3T3
cAMP conc. [µM]
+DMSO
+ISX_20
+ISX_40
+FSK
+SQ22536

- Figure S3 -

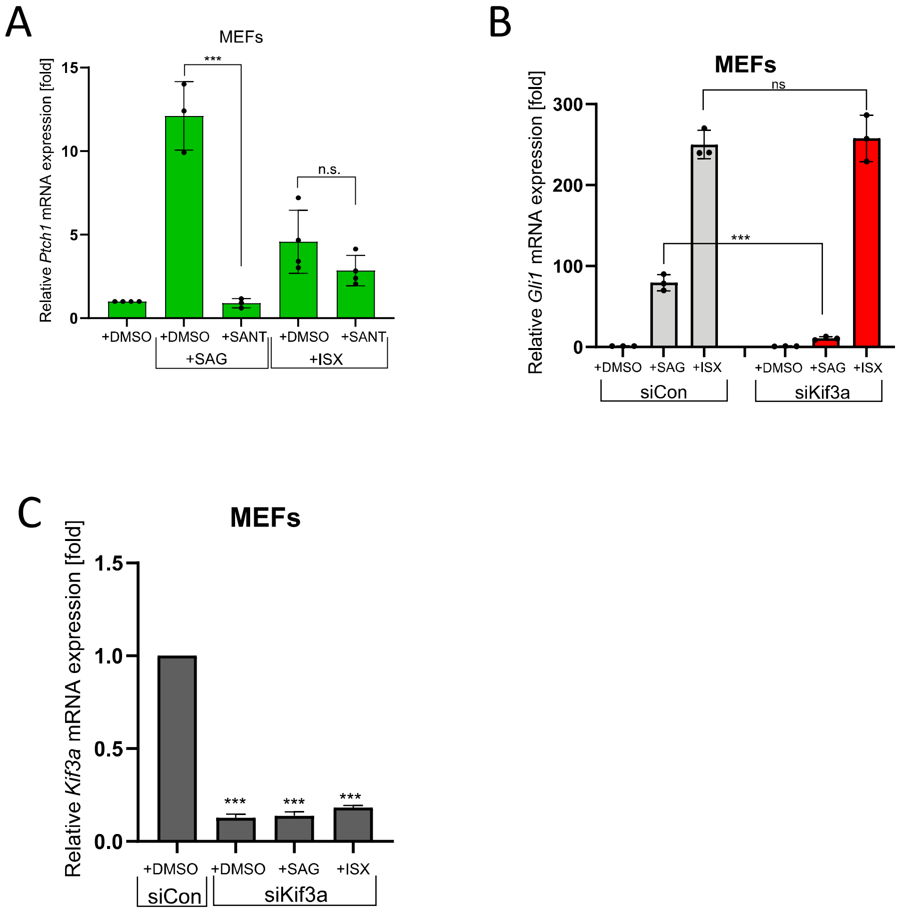
A
MEFs
Relative Ptch1 mRNA expression [fold]
15
10
5
0
***
n.s.
+DMSO
+DMSO
+SANT
+DMSO
+SANT
+SAG
+ISX
B
MEFs
Relative Gli1 mRNA expression [fold]
300
200
100
0
ns
***
+DMSO
+SAG
+ISX
+DMSO
+SAG
+ISX
siCon
siKif3a
C
MEFs
Relative Kif3a mRNA expression [fold]
1.5
1.0
0.5
0.0
***
***
***
+DMSO
+DMSO
+SAG
+ISX
siCon
siKif3a

- Figure S4 -

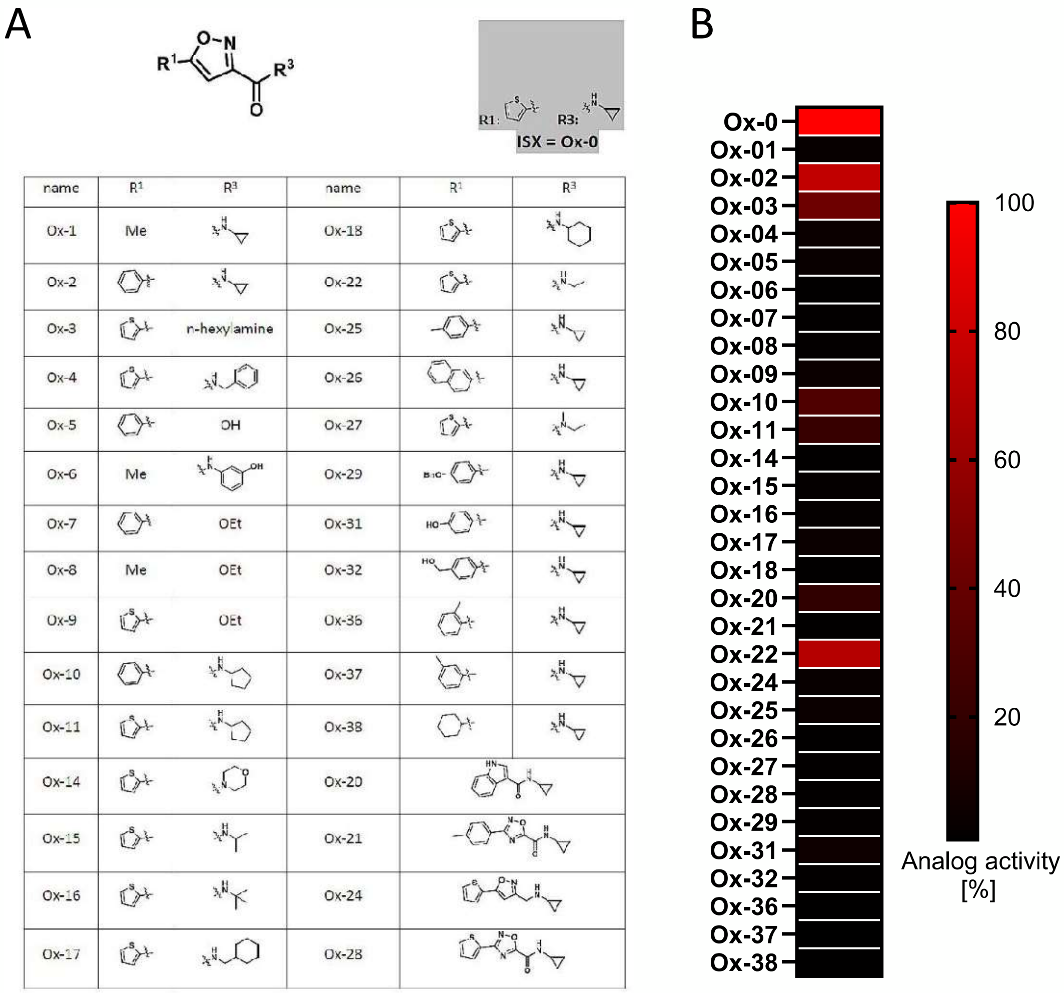
A
ISX = Ox-0
name
R1
R3
Ox-1
Me
Ox-2
Ox-3
n-hexylamine
Ox-4
Ox-5
OH
Ox-6
Me
Ox-7
OEt
Ox-8
Me
OEt
Ox-9
OEt
Ox-10
Ox-11
Ox-14
Ox-15
Ox-16
Ox-17
Ox-18
Ox-22
Ox-25
Ox-26
Ox-27
Ox-29
Ox-31
Ox-32
Ox-36
Ox-37
Ox-38
Ox-20
Ox-21
Ox-24
Ox-28
B
Ox-0
Ox-01
Ox-02
Ox-03
Ox-04
Ox-05
Ox-06
Ox-07
Ox-08
Ox-09
Ox-10
Ox-11
Ox-14
Ox-15
Ox-16
Ox-17
Ox-18
Ox-20
Ox-21
Ox-22
Ox-24
Ox-25
Ox-26
Ox-27
Ox-28
Ox-29
Ox-31
Ox-32
Ox-36
Ox-37
Ox-38
100
80
60
40
20
Analog activity
[%]

- Figure S5 -

A

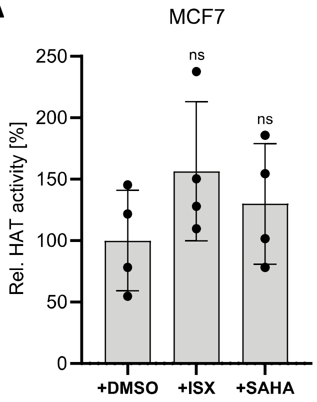

- Figure S6 -

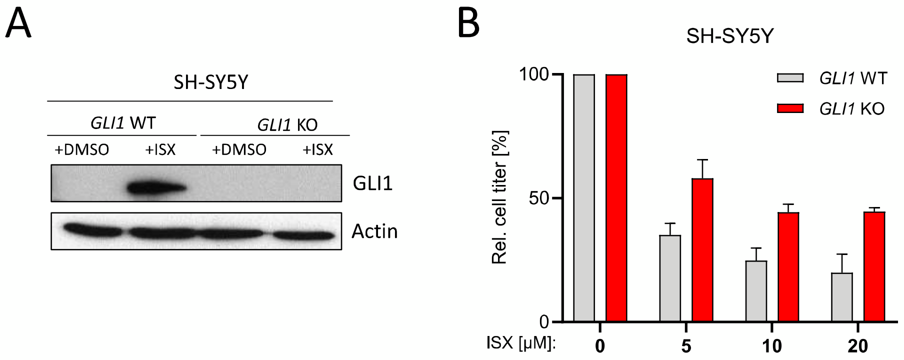
A
SH-SY5Y
GLI1 WT
GLI1 KO
+DMSO
+ISX
+DMSO
+ISX
GLI1
Actin
B
SH-SY5Y
GLI1 WT
GLI1 KO
Rel. cell titer [%]
100
50
0
ISX [µM]:
0
5
10
20

Koeniger et al.

Supplemental material

qPCR primer sequences:

human		
**Gene**	sequence (5´→3´)	
GLI1	hGLI1_qFor	TCTGGACATACCCCACCTCCCTCTG
	hGLI1_qRev	ACTGCAGCTCCCCCAATTTTTCTGG
GLI2	hGLI2_qFor	TGGCCGCTTCAGATGACAGATGTTG
	hGLI2_qRev	CGTTAGCCGAATGTCAGCCGTGAAG
GLI3	hGLI3_qFor	TGGACCCCAGGAATGGTTACATGGAG
	hGLI3_qRev	TGCAATGGAGGAATCGGAGATGGAT
PTCH1	hPTCH1_qFor	CCGCCTTCGCTCTGGAGCAGATT
	hPTCH1_qRev	TCTGAAACTTCGCTCTCAGCCACAGC
SMO	hSMO_qFor	CACGGCAAGCTCGTGCTCTGGT
	hSMO_qRev	GCTCCACCCGGTCATTCTCACACTT
MATN2	hMATN2_qFor	CGTGGCTACACTCTGGACCCCAATG
	hMATN2_qRev	AGCCTTCTGAGCACTGGCAGACGAA
KAL1	hKAL1_qFor	TACCGACTGGAAGTGCAAGTGCTGACC
	hKAL1_qRev	GGCTTGTAATGATGTGGATGACGATGC
VMAT2	hVMAT2_qFor	GCCTTCTCCAGCAGCTATGCCTTCC
	hVMAT2_qRev	CGATTCCCATGACGTTGCCTCTCTC
CDKN2A	hCDKN2A_qFor	ATAGTTACGGTCGGAGGCCGATCCA
	hCDKN2A_qRev	GGCATCTATGCGGGCATGGTTACTG
CDKN2B	hCDKN2B_qFor	GCGCTTTTTCCCAGAAGCAATCCAG
	hCDKN2B_qRev	CATTACCCTCCCGTCGTCCTTCTGC
CCNA2	hCCNA2_qFor	CAAAGCACCACAGCATGCACAACAG
	hCCNA2_qRev	CTGGTGGGTTGAGGAGAGAAACACCA
CCNB2	hCCNB2_qFor	AGCACATGGCCAAGAATGTGGTGAA
	hCCNB2_qRev	GGAGGCAAGGTCTTTGACGGCTTTT
CCND1	hCCND1_qFor	CACCTAGCAAGCTGCCGAACCAAAA
	hCCND1_qRev	TCACGACAGACAAAGCGTCCCTCAA
CCNE1	hCCNE1_qFor	AGGGAGACGGGGAGCTCAAAACTGA
	hCCNE1_qRev	CTTTGGTGGAGAAGGATGGGGTGGT
CCNE2	hCCNE2_qFor	GGCATTATGACACCACCGAAGAGCA
	hCCNE2_qRev	TTGGCTAGGGCAATCAATCACAGCA
RPLP0	mGli1_qFor	CCCATAGGGTCTCGGGGTCTCAAAT
	mGli1_qRev	GGAGGACCTGCGGCTGACTGTGTAA

qPCR primer sequences:

mouse

Gene		sequence (5´→3´)
Gli1	mGli1_qFor	CCCATAGGGTCTCGGGGTCTCAAAT
	mGli1_qRev	GGAGGACCTGCGGCTGACTGTGTAA
Gli2	mGli2_qFor	TGAGGAGAGTGTGGAGGCCAGTAGCA
	mGli2_qRev	CCGGGGCTGGACTGACAAAGC
Gli3	mGli3_qFor	AAAGCGGGAAGAGTGCCTCCAGGT
	mGli3_qRev	TGGCTGCTGCATGAAGACTGACCAC
Ptch1	mPtch1_qFor	CGCCTTCGCTCTGGAGCAGATTTC
	mPtch1_qRev	TGAGGAGACCCACAACCAAAAACTTGC
Ptch2	mPtch2_qFor	CCCGTGGTAATCCTCGTGGCCTCTAT
	mPtch2_qRev	TCCATCAGTCACAGGGGCAAAGGTC
Smo	mSmo_qFor	GAGGAGCCATATTGCCCCAGGATGT
	mSmo_qRev	TCCGGCCCAAACGCTTCTCTAACTC
Sufu	mSufu_qFor	GGAGCCCTCATCCCTCTCTGCCTAA
	mSufu_qRev	TACGGGTGTTCCTCAGTGGCAAAGG
Kif3a	mKif3a_qFor	CAAGGGGAAAGCAAGGCCAAAGATG
	mKif3a_qRev	CTCTCCAGGCATGGGACAGCACTCT
Rplp0	mP0_qFor	TGCACTCTCGCTTTCTGGAGGGTGT
	mP0_qRev	AATGCAGATGGATCAGCCAGGAAGG

siRNA sequences:

siRNA sequences (targeting mouse genes)

Name	Target sequence
siCon (Qiagen's All-Star; siAll)	AAUUCUCCGAACGUGUCACGU
siGli1_5	GAACUUCUGUGAUGGGCAA
siGli1_6	GUCCUAUUCACGCCUUGAA
siGli1_7	GGACUUUGUGGCUAUCCUA
siKif3a_1	CCUGAGACCGUAAUUGAUU
siKif3a_2	CGACUAAUAUGAACGAGCA
siKif3a_3	AGACUUAUCAGCAUAUGUA
siKif3a_4	CAUGAUGUGUGCAAAUAUU

Medicinal chemistry: Materials and methods

Compounds Ox-05, Ox-06, Ox-07, Ox-08, and Ox-09 are commercially available and were purchased from ABCR, Germany.

Compounds Ox-1, Ox-4, Ox-09, Ox-11, Ox-14, Ox-16, Ox-18, and Ox-28 have already been described elsewhere, however, full experimental details were not disclosed. [1]

General synthetic:

Unless stated otherwise, all commercially available starting materials and solvents were purchased and used without further purification. Microwave irradiation was applied with a Discover BenchMate Plus (CEM GmbH). Column chromatography was performed on prepacked flash chromatography columns (silica gel), thin-layer chromatography (TLC) on pre-coated TLC plates (silica gel 60 F254). ^{1}H and ^{13}C NMR spectra were recorded on a Jeol ECA-500 spectrometer. Chemical shifts (δ) are given in ppm with the residual solvent signal used as reference ($CDCl_3$: 7.26 / 77.16, DMSO-d_6: 2.50 / 39.52). Coupling constants (J) are reported in Hz. EI-mass spectra were recorded on an AccuTOF-GCv (JEOL) and ESI-mass spectra on an LTQ-FT (Finnigan). Elemental combustion analyses were performed on a Vario MICRO cube (Elementar Analysensysteme GmbH). Quantitative NMR (qNMR) measurements were recorded on a Jeol ECA-500 spectrometer using maleic acid, purchased from Sigma-Aldrich (99.94 % purity), as internal reference standard. Melting points were determined using a melting point meter M 5000 (Krüss) or an MPM-M2 (Schorpp) and are uncorrected.

General procedure A for the synthesis of Ox-01, Ox-02, Ox-03, Ox-04, Ox-10, Ox-11, Ox-14, Ox-15, Ox-16, utilizing the corresponding acid chlorides, which were prepared in situ as outlined in scheme 1.

The respective carboxylic acid (1.00 eq.) was suspended in DCM and DMF (1 drop), then excessive $SOCl_2$ was added and the mixture heated to reflux for 3 h. Subsequently, cylo hexane (*c*Hex) was added and the mixture concentrated *in vacuo*. The crude product was immediately submitted to the next reaction step without further purification.

The respective amine (1.10 eq.) and Et_3N (5 eq.) were dissolved in DCM at room temperature, followed by the drop-wise addition of the crude acid chloride (1.00 eq.) in DCM and stirring was continued overnight. Then the mixture was quenched with water, the organic phase washed with HCl (1 M, 3x), followed by a saturated aqueous solution of NaCl, dried over $MgSO_4$, and finally concentrated *in vacuo* to afford the respective product as colorless crystals.

Scheme 1:

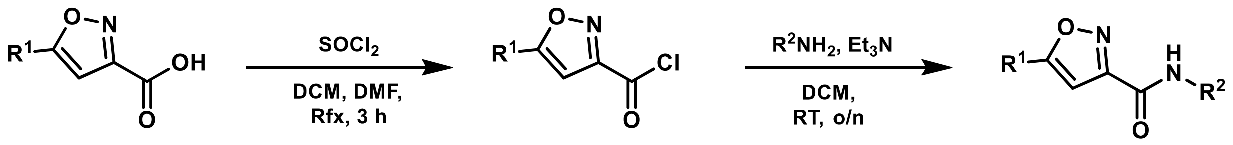

General procedure B for the synthesis of Ox-12, Ox-17, Ox-18, Ox-22, Ox-24, Ox-25, Ox-26, Ox-27, Ox-29, Ox-31, Ox-32, Ox-36, Ox-37, Ox-38 following the synthetic route depicted in Scheme 2.

Scheme 2:

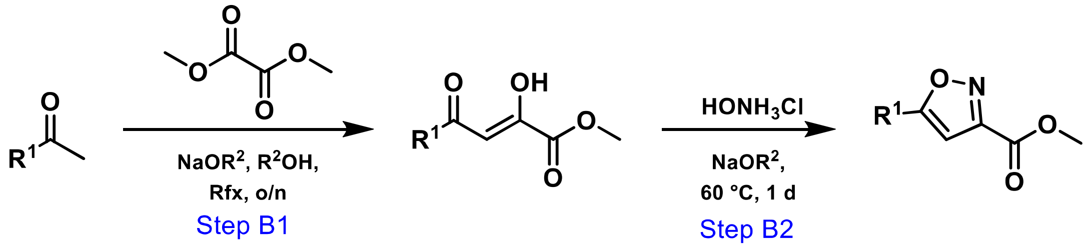

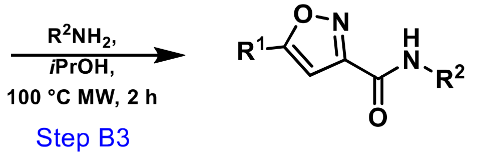

Step B1: Synthesis of 2-hydroxy-4-oxo-but-2-enoates

method A:

To a freshly prepared 0.79 M solution of Na in MeOH (2.00 eq.), a solution of the respective ketone (1.00 eq.) and dimethyl oxalate (2.00 eq.) in MeOH (2.0 M based on ketone) was added and the reaction mixture was refluxed overnight. Then aqueous HCl was added and MeOH was removed *in vacuo*. Either, the resulting precipitate was separated by filtration and dried *in vacuo* (**A1**) or the aqueous phase extracted with DCM. The combined organic phase was washed with a saturated aqueous solution of NaCl, dried over $MgSO_4$ and concentrated *in vacuo* (**A2**). The crude product was immediately submitted to the next reaction step.

method B:

The respective ketone (1.00 eq.) and dimethyl oxalate (1.00 eq.) were dissolved in dry Et_2O (1 M) and a freshly prepared solution of Na (1.20 eq., in MeOH (0.2 M)) was added slowly. The reaction mixture was stirred at rt overnight, the precipitate was separated by filtration, washed with cold MeOH, then with Et_2O, and finally dried *in vacuo.*

The enolate was redissolved in H_2O and stirred at rt for 1 h. Subsequently, the solution was acidified to pH 3 with conc. AcOH and the mixture stirred for 1 h at 0 °C. The resulting precipitate was filtered, washed with H_2O and dried *in vacuo* to afford the product as an off-white solid, which was immediately

submitted to the next reaction step.

Step B2: Synthesis of isoxazoles

To a solution of the respective crude 2-hydroxy-4-oxo-but-2-enoates (1.00 eq., e.g. from **step B1 method A** or **B**) in MeOH (0.3 M) NH_2OH^*HCl (1.18 eq.) was added and the resulting suspension stirred for 24 h at 60 °C. Subsequently, the reaction mixture was cooled to rt, concentrated *in vacuo*, redissolved in HCl (1 M) and then extracted with EtOAc. The combined organic phase was washed with a saturated aqueous solution of NaCl, dried over $MgSO_4$ and concentrated *in vacuo*. Purification was carried out *via* column chromatography to afford the respective product as colorless crystals. [2]

Step B3: Synthesis of isoxazole carboxamides from their corresponding methyl esters

The respective amine (2.00 eq.) and methyl ester (1.00 mmol, 1.00 eq., e.g. from **step B2**) were suspended in *i*-PrOH (1.0 M), submitted to a microwave device and stirred for 2 h at 100 °C (300 W). The precipitate was filtered, washed with *i*-PrOH and dried *in vacuo* to afford the respective product as colorless solid.

<u>*N*-Cyclopropyl-5-(thiophen-2-yl)isoxazol-3-carboxamide (Ox-0)</u>

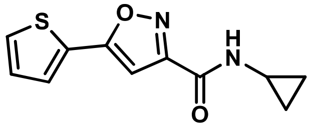

Following general procedure B, step B3 reaction of the respective methyl ester (2000 mg, 9.56 mmol) and cyclopropylamine (1.33 mL, 19.10 mmol, 2 eq.) rendered **Ox-12** (1300 mg, 5.55 mmol) in 58 % overall yield : ^{1}H-NMR (500 MHz, DMSO-d$_6$): δ_H = 8.83 (d, $^3J_{H,H}$ = 4.4 Hz, 1H), 7.86 (dd, $^3J_{H,H}$ = 5.0 Hz, $^4J_{H,H}$ = 1.1 Hz, 1H), 7.78 (dd, $^3J_{H,H}$ = 3.7 Hz, $^4J_{H,H}$ = 1.0 Hz, 1H), 7.26 (dd, $^3J_{H,H}$ = 5.0 Hz, 3.7 Hz, 1H), 7.20 (s, 1H), 2.90 – 2.83 (m, 1H), 0.75 – 0.58 (m, 4H); ^{13}C-NMR (125 MHz, DMSO-d$_6$): δ_C = 165.5, 159.5, 159.3, 130.0, 128.7, 128.6, 127.5, 99.0, 22.7, 5.6; HRMS (ESI, *m/z*): [M+Na]$^+$ calculated for $[C_{11}H_{10}N_2O_2NaS]^+$: 257.0355, found: 257.0356; combustion analysis (C/H/N, *%*): calculated for $[C_{11}H_{10}N_2O_2S]$: 56.40 / 4.30 / 11.96, found: 56.49 / 4.35 / 11.95; mp: 154 °C.

<u>*N*-Cyclopropyl-5-methylisoxazol-3-carboxamide (Ox-01)</u>

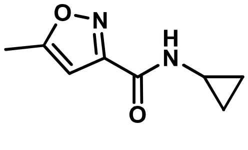

N-Cyclopropyl-5-methylisoxazol-3-carboxamide was prepared according to general procedure A employing cyclopropylamine (0.15 M solution, 7.33 mL, 1.10 eq.) and the corresponding acid chloride (145 mg, 1.00 mmol, 0.25 M, 1.00 eq.). The obtained product was further purified via column

chromatography (*c*Hex / EtOAc, 2:1) yielding **Ox-01** (90 mg, 0.54 mmol) in 54 % overall yield: ^{1}H-NMR (500 MHz, DMSO-d$_6$): δ_H = 8.69 (d, $^3J_{H,H}$ = 2.5 Hz, 1H), 6.50 (s, 1H), 2.88 – 2.78 (m, 1H), 2.44 (s, 3H), 0.73 – 0.55 (m, 4H); ^{13}C-NMR (125 MHz, DMSO-d$_6$): δ_C = 171.5, 160.4, 159.4, 101.7, 23.2, 12.3, 6.1; MS (ESI, *m/z*): [M+NH$_4$]$^+$ calculated for [$C_8H_{14}N_3O_2$]$^+$: 184.11, found: 184.16; combustion analysis (C/H/N, *%*): calculated for [$C_8H_{10}N_2O_2$]: 57.82 / 6.07 / 16.86, found: 58.20 / 6.09 / 16.99.

***N*-Cyclopropyl-5-phenylisoxazol-3-carboxamide (Ox-02)**

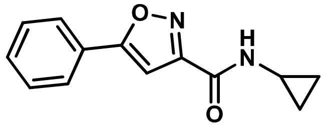

N-Cyclopropyl-5-phenylisoxazol-3-carboxamide was prepared according to general procedure A employing cyclopropylamine (0.15 M solution, 5.13 mL, 1.10 eq.) and the corresponding acid chloride (145 mg, 0.70 mmol, 0.18 M). Column chromatography (DCM / MeOH, 20:1) rendered **Ox-02** (90 mg, 0.39 mmol) in 56 % overall yield: ^{1}H-NMR (500 MHz, DMSO-d$_6$): δ_H = 8.68 (d, $^3J_{H,H}$ = 7.3 Hz, 1H), 7.95 – 7.89 (m, 2H), 7.60 – 7.48 (m, 3H), 7.33 (s, 1H), 4.29 – 4.17 (m, 1H), 1.97 – 1.45 (m, 4H); ^{13}C-NMR (125 MHz, DMSO-d$_6$): δ_C = 170.1, 159.9, 158.2, 130.8, 129.4, 126.4, 125.8, 99.9, 22.8, 5.7; MS (ESI, *m/z*): [M+NH$_4$]$^+$ calculated for [$C_{13}H_{16}N_3O_2$]$^+$: 246.12, found: 246.22; combustion analysis (C/H/N, *%*): calculated for [$C_{13}H_{12}N_2O_2$]: 68.41 / 5.30 / 12.27, found: 68.19 / 5.36 / 12.40; mp: 139 °C.

***N*-Hexyl-5-(thiophen-2-yl)isoxazol-3-carboxamide (Ox-03)**

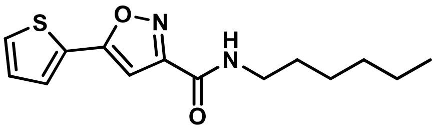

N-Hexyl-5-(thiophen-2-yl)isoxazol-3-carboxamide was prepared according to general procedure A employing *n*-hexylamine (0.17 M solution, 4.85 mL, 1.10 eq.) and the corresponding acid chloride (160 mg, 0.75 mmol, 0.15 M). Column chromatography (*c*Hex / EtOAc, 6:1) gave rise to **Ox-03** (197 mg, 0.71 mmol) in 94 % overall yield: ^{1}H-NMR (500 MHz, CDCl$_3$): δ_H = 8.75 (t, $^3J_{H,H}$ = 5.6 Hz, 1H), 7.86 (d, $^3J_{H,H}$ = 5.0 Hz, 1H), 7.78 (d, $^3J_{H,H}$ = 3.7 Hz, 1H), 7.26 (dd, $^3J_{H,H}$ = 4.7 Hz, 4.0 Hz, 1H), 7.16 (s, 1H), 3.24 (q, $^3J_{H,H}$ = 6.7 Hz, 2H), 1.62 – 1.44 (m, 2H), 1.37 – 1.19 (m, 6H), 0.97 – 0.79 (m, 3H); ^{13}C-NMR (125 MHz, CDCl$_3$): δ_C = 166.6, 159.4, 158.7, 128.8, 128.6, 128.3, 127.8, 99.0, 39.7, 31.6, 29.5, 26.7, 22.7, 14.2; MS (ESI, *m/z*): [M+NH$_4$]$^+$ calculated for [$C_{14}H_{22}N_3O_2S$]$^+$: 296.14, found: 296.25; combustion analysis (C/H/N, *%*): calculated for [$C_{14}H_{18}N_2O_2S$] * 0.25 H_2O: 59.44 / 6.59 / 9.90, found: 59.27 / 6.41 / 9.85; mp: 92 °C.

***N*-Benzyl-5-(thiophen-2-yl)isoxazole-3-carboxamide (Ox-04)**

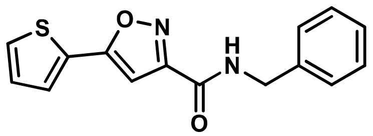

N-Benzyl-5-(thiophen-2-yl)isoxazole-3-carboxamide was prepared according to general procedure A employing the corresponding acid chloride (160 mg, 0.75 mmol, 0.13 M) and benzylamine (0.18 M, 4.58 mL, 1.10 eq.). Column chromatography (*c*Hex / EtOAc, 4:1) rendered **Ox-04** (70 mg, 0.25 mmol) in 94 % overall yield: ^{1}H-NMR (500 MHz, DMSO-d$_6$): δ_H = 9.35 (t, $^3J_{H,H}$ = 6.1 Hz, 1H), 7.87 (dd, $^3J_{H,H}$ = 5.0 Hz, $^4J_{H,H}$ = 1.1 Hz, 1H), 7.79 (dd, $^3J_{H,H}$ = 3.6 Hz, $^4J_{H,H}$ = 1.1 Hz, 1H), 7.38 – 7.34 (m, 4H), 7.30 – 7.23 (m, 2H), 7.20 (s, 1H), 4.47 (d, $^3J_{H,H}$ = 6.2 Hz, 2H); ^{13}C-NMR (125 MHz, DMSO-d$_6$): δ_C = 166.3, 160.1, 158.8, 139.4, 130.7, 129.30, 129.28, 128.9, 128.1, 127.9, 127.5, 99.7, 42.9; MS (ESI, *m/z*): $[M+NH_4]^+$ calculated for $[C_{15}H_{16}N_3O_2S]^+$: 302.10, found: 302.19; combustion analysis (C/H/N, *%*): calculated for $[C_{15}H_{12}N_2O_2S]$ * 0.5 H_2O: 61.42 / 4.47 / 9.55, found: 61.56 / 4.24 / 9.52; mp: 135 °C.

***N*-Cyclopentyl-5-phenylisoxazol-3-carboxamide (Ox-10)**

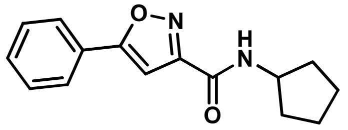

In accordance with general procedure A, *N*-cyclopentyl-5-phenylisoxazol-3-carboxamide was prepared utilizing the respective acid chloride (270 mg, 1.30 mmol, 0.16 M) and cyclopentylamine (0.20 M, 7.15 mL, 1.10 eq.). Purification via column chromatography (*c*Hex / EtOAc, 9:1) gave rise to **Ox-10** (237 mg, 0.92 mmol) in 71 % overall yield: ^{1}H-NMR (500 MHz, DMSO-d$_6$): δ_H = 8.68 (d, $^3J_{H,H}$ = 7.3 Hz, 1H), 7.95 – 7.89 (m, 2H), 7.60 – 7.48 (m, 3H), 7.33 (s, 1H), 4.23 (sm, 1H), 1.97 – 1.45 (m, 8H); ^{13}C-NMR (125 MHz, DMSO-d$_6$): δ_C = 170.1, 159.9, 158.2, 130.8, 129.4, 126.4, 125.8, 99.9, 50.7, 31.9, 23.6; HRMS (ESI, *m/z*): $[M+Na]^+$ calculated for $[C_{15}H_{16}N_2O_2Na]^+$: 279.1104, found: 279.1106; combustion analysis (C/H/N, *%*): calculated for $[C_{15}H_{16}N_2O_2]$: 70.29 / 6.29 / 10.93, found: 70.20 / 6.30 / 10.89; mp: 139 °C.

***N*-Cyclopentyl-5-(thiophen-2-yl)isoxazol-3-carboxamide (Ox-11)**

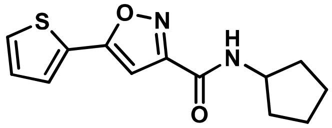

According to general procedure A, utilization of the respective acid chloride (147 mg, 0.69 mmol, 1.00 eq.), cyclopentylamine (0.08 mL, 1.20 mmol, 1.75 eq.), and Et_3N (2.48 eq.) gave rise to **Ox-11** (131 mg, 0.50 mmol) in 73 % overall yield after column chromatography (*c*Hex / EtOAc, 9:1): ^{1}H-NMR (500 MHz, DMSO-d$_6$): δ_H = 8.68 (d, $^3J_{H,H}$ = 7.6 Hz, 1H), 7.86 (dd, $^3J_{H,H}$ = 5.0 Hz, $^4J_{H,H}$ = 1.1 Hz, 1H), 7.77 (dd, $^3J_{H,H}$ = 3.7

Hz, $^4J_{H,H}$ = 1.1 Hz, 1H), 7.26 (dd, $^3J_{H,H}$ = 5.0 Hz, 3.7 Hz, 1H), 7.16 (s, 1H), 4.28 – 4.15 (m, 1H), 1.96 – 1.44 (m, 8H); ^{13}C-NMR (125 MHz, DMSO-d$_6$): δ_C = 165.4, 159.7, 157.8, 129.9, 128.7, 128.5, 127.6, 99.1, 50.7, 31.8, 23.5; HRMS (ESI, *m/z*): [M+Na]$^+$ calculated for [$C_{13}H_{14}N_2O_2NaS$]$^+$: 285.0668, found: 285.0670; combustion analysis (C/H/N, *%*): calculated for [$C_{13}H_{14}N_2O_2S$]: 59.52 / 5.38 / 10.68, found: 59.33 / 5.39 / 10.61; mp: 148 °C.

Morpholino(5-(thiophen-2-yl)isoxazol-3-yl)methanon (Ox-14)

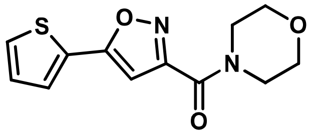

Utilization of the respective acid chloride (217 mg, 1.02 mmol, 0.10 M) and morpholine (0.06 M, 18.7 mL, 1.1 eq.) according to general procedure A followed by column chromatography (*c*Hex / EtOAc, 6:1) and re-crystallization (*c*Hex) gave rise to **Ox-14** (60 mg, 0.23 mmol) in 22 % overall yield: ^{1}H-NMR (500 MHz, DMSO-d$_6$): δ_H = 7.87 (dd, $^3J_{H,H}$ = 4.9 Hz, $^4J_{H,H}$ = 1.2 Hz, 1H), 7.78 (dd, $^3J_{H,H}$ = 3.7 Hz, $^4J_{H,H}$ = 1.2 Hz, 1H), 7.27 (dd, $^3J_{H,H}$ = 5.0 Hz, 3.6 Hz, 1H), 7.11 (s, 1H), 3.69 – 3.58 (m, 8H); ^{13}C-NMR (125 MHz, DMSO-d$_6$): δ_C = 164.9, 158.81, 158.78, 130.1, 128.7, 127.4, 100.0, 66.3, 65.8, 47.0, 42.2; HRMS (ESI, *m/z*): [M+Na]$^+$ calculated for [$C_{12}H_{12}N_2O_3NaS$]$^+$: 287.0461, found: 287.0459; combustion analysis (C/H/N, *%*): calculated for [$C_{12}H_{12}N_2O_3S$]: 54.53 / 4.58 / 10.60, found: 54.51 / 4.65 / 10.61; mp: 127 °C.

***N*-Isopropyl-5-(thiophen-2-yl)isoxazole-3-carboxamide (OX-15)**

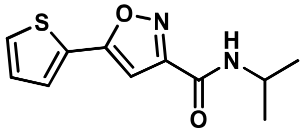

In accordance to general procedure A, **Ox-15** was obtained employing the respective acid chloride (213 mg, 1.00 mmol, 1.0 eq) and *i*-propylamine (2.07 mmol, 0.17 mL, 2.07 eq.). Column chromatography (*c*Hex / EtOAc, 6:1) rendered **Ox-15** (139 mg, 0.59 mmol) in 59 % yield over two steps: ^{1}H-NMR (500 MHz, DMSO-d$_6$): δ_H = 8.58 (d, $^3J_{H,H}$ = 8.0 Hz, 1H), 7.86 (dd, $^3J_{H,H}$ = 4.9 Hz, $^4J_{H,H}$ = 1.2 Hz, 1H), 7.78 (dd, $^3J_{H,H}$ = 3.6 Hz, $^4J_{H,H}$ = 1.3 Hz, 1H), 7.26 (dd, $^3J_{H,H}$ = 4.9 Hz, 3.7 Hz, 1H), 7.16 (s, 1H), 4.10 (sm, 1H), 1.17 (d, $^3J_{H,H}$ = 6.6 Hz, 6H); ^{13}C-NMR (125 MHz, DMSO-d$_6$): δ_C = 165.4, 159.8, 157.3, 129.9, 128.7, 128.5, 127.6, 99.1, 99.0, 41.0, 21.9; HRMS (ESI, *m/z*): [M+Na]$^+$ calculated for [$C_{11}H_{12}N_2O_2NaS$]$^+$: 259.0512, found: 259.0509; combustion analysis (C/H/N, *%*): calculated for [$C_{11}H_{12}N_2O_2S$]: 55.92 / 5.12 / 11.86, found: 55.88 / 5.13 / 11.88; mp: 112 °C.

N-(tert-Butyl)-5-(thiophen-2-yl)isoxazol-3-carboxamide (Ox 16)

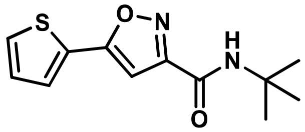

In accordance with general procedure A, *N*-(*tert*-butyl)-5-(thiophen-2-yl)isoxazol-3-carboxamide was prepared utilizing the respective acid chloride (162 mg, 0.76 mmol, 0.10 M) and *tert.*-butylamine (0.17 M, 4.92 mL, 1.10 eq.). Purification via column chromatography (*c*Hex / EtOAc, 6:1) gave rise to **Ox-16** (51 mg, 0.20 mmol) in 27 % overall yield: ^{1}H-NMR (500 MHz, DMSO-d$_6$): δ_H = 7.99 (br s, 1H), 7.85 (dd, $^3J_{H,H}$ = 5.2 Hz, $^4J_{H,H}$ = 1.2 Hz, 1H), 7.76 (dd, $^3J_{H,H}$ = 3.6 Hz, $^4J_{H,H}$ = 1.3 Hz, 1H), 7.26 (dd, $^3J_{H,H}$ = 5.2 Hz, 3.7 Hz, 1H), 7.13 (s, 1H), 1.38 (s, 9H); ^{13}C-NMR (125 MHz, DMSO-d$_6$): δ_C = 165.3, 160.3, 157.9, 129.9, 128.7, 128.5, 127.6, 99.0, 51.3, 28.3; HRMS (ESI, *m/z*): $[M+Na]^+$ calculated for $[C_{12}H_{14}N_2O_2NaS]^+$: 273.0668, found: 273.0666; combustion analysis (C/H/N, *%*): calculated for $[C_{12}H_{14}N_2O_2S]$: 57.58 / 5.64 / 11.19, found: 57.59 / 5.64 / 11.21; mp: 119 °C.

Methyl 5-(thiophen-2-yl)isoxazole-3-carboxylate

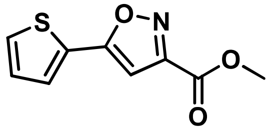

Following general procedure B 1, reaction of 1-(thiophen-2-yl)ethan-1-on (4.28 mL, 39.63 mmol) provided intermediate methyl-(*Z*)-2-hydroxy-4-oxo-4-(thiophen-2-yl)but-2-enoate, 6450 mg (30.39 mmol) of which were reacted with NH_2OH^*HCl (1.18 eq.) in the following step (B2) giving rise to methyl-5-(thiophen-2-yl)isoxazol-3-carboxylate (4990 mg, 23.85 mmol) after column chromatography (DCM/MeOH, 20:1) in 60 % overall yield: ^{1}H-NMR (500 MHz, DMSO-d$_6$): δ_H = 7.89 (dd, $^3J_{H,H}$ = 5.0 Hz, $^4J_{H,H}$ = 1.3 Hz, 1H), 7.83 (dd, $^3J_{H,H}$ = 3.7 Hz, $^4J_{H,H}$ = 1.2 Hz, 1H), 7.32 (s, 1H), 7.28 (dd, $^3J_{H,H}$ = 5.2 Hz, 3.7 Hz, 1H), 3.92 (s, 3H); ^{13}C-NMR (125 MHz, DMSO-d$_6$): δ_C = 166.4, 159.6, 156.6, 130.4, 129.0, 128.7, 127.2, 99.9, 52.8; HRMS (ESI, *m/z*): $[M+H]^+$ calculated for $[C_9H_8NO_3S]^+$: 210.0219, found: 210.0222; combustion analysis (C/H/N/S, *%*): calculated for $[C_9H_7NO_3S]$: 51.67 / 3.37 / 6.69 / 15.32, found: 51.59 / 3.39 / 6.66 / 15.27.

N-(Cyclohexylmethyl)-5-(thiophen-2-yl)isoxazol-3-carboxamide (Ox-17)

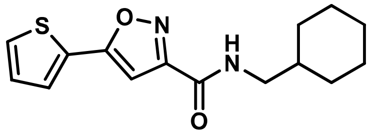

According to general procedure B, step B3, utilization of the respective methyl ester (209 mg, 1.0 mmol, 1.00 eq.) and cyclohexylmethanamine (0.26 mL, 2.00 mmol, 2.00 eq.) gave rise to **Ox-17** (188 mg, 0.75 mmol) as colorless crystals in 75 % overall yield: ^{1}H-NMR (500 MHz, DMSO-d$_6$): δ_H = 8.56 (t, $^{3}J_{H,H}$ = 5.9 Hz, 1H), 7.86 (dd, $^{3}J_{H,H}$ = 4.9 Hz, $^{4}J_{H,H}$ = 1.2 Hz, 1H), 7.78 (dd, $^{3}J_{H,H}$ = 3.7 Hz, $^{4}J_{H,H}$ = 1.2 Hz, 1H), 7.26 (dd, $^{3}J_{H,H}$ = 4.9 Hz, 3.7 Hz, 1H), 7.16 (s, 1H), 3.10 (dd, $^{3}J_{H,H}$ = 6.6 Hz, 6.6 Hz, 2H), 1.76 – 0.84 (m, 11H); ^{13}C-NMR (125 MHz, DMSO-d$_6$): δ_C = 165.5, 159.7, 158.2, 129.9, 128.6, 128.5, 127.6, 99.0, 45.0, 37.2, 30.3, 26.0, 25.3; HRMS (ESI, *m/z*): [M+Na]$^+$ calculated for [$C_{15}H_{18}N_2O_2NaS$]$^+$: 313.0981, found: 313.0983; combustion analysis (C/H/N, *%*): calculated for [$C_{15}H_{18}N_2O_2S$]: 62.04 / 6.25 / 9.65, found: 62.11 / 6.50 / 9.50; mp: 156 °C.

***N*-Cyclohexyl-5-(thiophen-2-yl)isoxazol-3-carboxamide (Ox-18)**

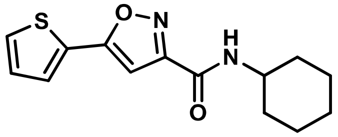

Following general procedure B step B3, utilization of the respective methyl ester (209 mg, 1.00 mmol, 1.00 eq.) and cyclohexylamine (0.23 mL, 2.0 mmol, 2.00 eq.) furnished **Ox-18** (188 mg, 0.68 mmol) as colorless crystals in 68 % overall yield: ^{1}H-NMR (500 MHz, DMSO-d$_6$): δ_H = 8.56 (d, $^{3}J_{H,H}$ = 8.0 Hz, 1H), 7.86 (dd, $^{3}J_{H,H}$ = 4.9 Hz, $^{4}J_{H,H}$ = 1.2 Hz, 1H), 7.78 (dd, $^{3}J_{H,H}$ = 3.7 Hz, $^{4}J_{H,H}$ = 1.2 Hz, 1H), 7.23 (dd, $^{3}J_{H,H}$ = 4.9 Hz, 3.7 Hz, 1H), 7.16 (s, 1H), 3.80 – 3.71 (m, 1H), 1.86 – 1.06 (m, 10H); ^{13}C-NMR (125 MHz, DMSO-d$_6$): δ_C = 165.4, 159.8, 157.3, 129.9, 128.6, 128.5, 127.6, 99.1, 48.2, 31.9, 25.0, 24.7; HRMS (ESI, *m/z*): [M+Na]$^+$ calculated for [$C_{14}H_{16}N_2O_2NaS$]$^+$: 299.0825, found: 299.0826; combustion analysis (C/H/N/S, *%*): calculated for [$C_{14}H_{16}N_2O_2S$]: 60.85 / 5.84 / 10.14 / 11.60, found: 60.46 / 5.78 / 10.04 / 11.30; mp: 180 °C.

***N*-Cyclopropyl-1*H*-indole-3-carboxamide (Ox-20)**

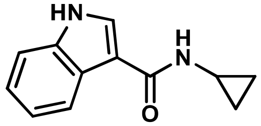

N-Cyclopropyl-1*H*-indole-3-carboxamide was prepared according to general procedure A employing cyclopropylamine (0.5 mL, 6.83 mmol, 1.10 eq.) and the corresponding acid chloride (1115 mg, 6.21 mmol, 0.31 M, 1.00 eq.). After stirring for 2 hours, the resulting precipitate was collected by filtration, washed with water, subsequently with *i*PrOH, and dried *in vacuo* to afford **Ox-20** as a colorless solid (412 mg, 2.06 mmol) in 33 % yield: ^{1}H-NMR (500 MHz, DMSO-d$_6$): δ_H = 11.47 (br s, 1H), 8.14 (d, $^{3}J_{H,H}$ = 8.0 Hz, 1H), 7.96 (d, $^{4}J_{H,H}$ = 2.9 Hz, 1H), 7.86 (d, $^{4}J_{H,H}$ = 3.2 Hz, 1H), 7.40 (d, $^{3}J_{H,H}$ = 8.0 Hz, 1H), 7.13 (td,

$^{3}J_{H,H}$ = 7.5 Hz, $^{4}J_{H,H}$ = 1.3 Hz, 1H), 7.09 (td, $^{3}J_{H,H}$ = 7.4 Hz, $^{4}J_{H,H}$ = 1.1 Hz, 1H), 2.84 – 2.76 (m, 1H), 0.69 – 0.51 (m, 4H); ^{13}C-NMR (125 MHz, DMSO-d$_6$): 165.8, 136.0, 127.5, 126.1, 121.7, 121.0, 120.2, 111.6, 110.5, 22.3, 5.9; HRMS (ESI, *m/z*): [M+Na]$^+$ calculated for [$C_{12}H_{12}N_2ONa$]$^+$: 223.0842, found: 223.0843; purity (qNMR, 500 MHz, DMSO-d$_6$, maleic acid): 94 %; mp: 209 °C.

<u>*N*-Cyclopropyl-3-(*p*-tolyl)-1,2,4-oxadiazole-5-carboxamide (Ox-21)</u>

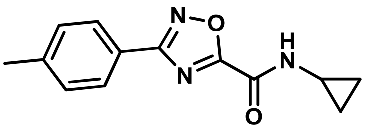

N-Hydroxy-4-methylbenzimidamide (314 mg, 2.09 mmol, 1.00 eq.), methyl 2-chloro-2-oxoacetate (0.58 mL, 6.27 mmol, 3.00 eq.) and K_2CO_3 (2.00 eq.) were suspended in MeCN (0.5 M) under an argon-atmosphere, submitted to a microwave device and stirred for 1 h at 120 °C (150 W). The reaction mixture was filtered, concentrated under reduced pressure and finally extracted with EtOAc. The combined organic phase was washed with a saturated aqueous solution of NaCl, dried over $MgSO_4$ and concentrated *in vacuo*. Column chromatography (dichloromethane) gave rise to methyl 3-(p-tolyl)-1,2,4-oxadiazole-5-carboxylate (372 mg, 1.70 mmol) as a colourless solid in 81% yield:
^{1}H-NMR (500 MHz, CDCl$_3$): δ_H = 8.03 (d, $^{3}J_{H,H}$ = 8.3 Hz, 2H), 7.31 (d, $^{3}J_{H,H}$ = 8.0 Hz, 2H), 4.10 (s, 3H), 2.42 (s, 3H); ^{13}C-NMR (125 MHz, CDCl$_3$): 169.6, 166.3, 154.8, 142.6, 129.9, 127.7, 122.9, 54.3, 21.8; HRMS (ESI, *m/z*): [M+Na]$^+$ calculated for [$C_{11}H_{11}N_2O_3$]: 219.0764, found: 219.0765; combustion analysis (C/H/N, *%*): calculated for [$C_{11}H_{10}N_2O_3$ *0.25 H_2O]: 59.32 / 12.58 / 4.75, found: 59.69 / 12.62 / 4.75; mp: 94 °C. Following general procedure B, step B3, reaction of the methyl ester (218 mg, 1.00 mmol, 1.00 eq) and cyclopropylamine (0.14 mL, 2.00 mmol, 2 eq.) rendered **Ox-21** (202 mg, 0.83 mmol) as colourless crystals in 83 % yield: ^{1}H-NMR (500 MHz, DMSO-d$_6$): δ_H = 9.46 (d, $^{3}J_{H,H}$ = 2.6 Hz, 1H), 7.94 (d, $^{3}J_{H,H}$ = 8.0 Hz, 2H), 7.93 (dd, $^{3}J_{H,H}$ = 8.6 Hz, $^{4}J_{H,H}$ = 0.6 Hz, 2H), 2.96 – 2.88 (m, 1H), 2.39 (s, 3H), 0.79 – 0.70 (m, 4H); ^{13}C-NMR (125 MHz, DMSO-d$_6$): 169.1, 168.0, 154.0, 142.0, 129.8, 127.1, 122.7, 23.1, 21.0, 5.6; HRMS (ESI, *m/z*): [M+Na]$^+$ calculated for for [$C_{13}H_{13}N_3O_2Na$]: 266.0900, found: 266.0903; combustion analysis (C/H/N, *%*): calculated for [$C_{13}H_{13}N_3O_2$]: 64.19 / 5.39 / 17.27, found: 64.16 / 5.37 / 17.36; mp: 132 °C.

<u>*N*-Ethyl-5-(thiophen-2-yl)isoxazol-3-carboxamide (Ox-22)</u>

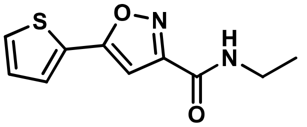

Ox-22 (127 mg, 0.57 mmol) was obtained as colorless crystals in 57 % overall yield employing ethylamine (60 % v/v in water, 0.17 mL, 2.00 eq.) and the respective methyl ester (209 mg, 1.00 mmol, 1.00 eq.) according to general procedure B, step B3: ^{1}H-NMR (500 MHz, DMSO-d$_6$): δ_H = 8.77 (t, $^{3}J_{H,H}$ = 5.3 Hz, 1H), 7.86 (dd, $^{3}J_{H,H}$ = 5.1 Hz, $^{4}J_{H,H}$ = 1.3 Hz, 1H), 7.78 (dd, $^{3}J_{H,H}$ = 3.6 Hz, $^{4}J_{H,H}$ = 1.3 Hz, 1H), 7.26 (dd, $^{3}J_{H,H}$ = 4.9 Hz, 3.7 Hz, 1H), 7.16 (s, 1H), 3.29 (dq, $^{3}J_{H,H}$ = 7.2 Hz, 6.0 Hz, 2H, overlaid by H_2O signal), 1.12 (t, $^{3}J_{H,H}$ = 7.2 Hz, 3H); ^{13}C-NMR (125 MHz, DMSO-d$_6$): δ_C = 165.5, 159.7, 157.9, 130.0, 128.7, 128.6, 127.5, 99.0, 33.7, 14.4; HRMS (ESI, *m/z*): [M+Na]$^+$ calculated for $[C_{10}H_{10}N_2O_2NaS]^+$: 245.0355, found: 245.0356; combustion analysis (C/H/N, *%*): calculated for $[C_{10}H_{10}N_2O_2S]$: 54.04 / 4.54 / 12.60, found: 53.95 / 4.51 / 12.58; mp: 125 °C.

***N*-((5-(Thiophen-2-yl)isoxazol-3-yl)methyl)cyclopropanamin (Ox-24)**

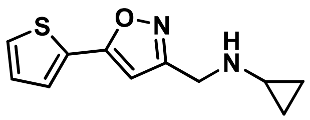

To an ice-cooled solution of methyl-5-(thiophen-2-yl)isoxazol-3-carboxylate (512 mg, 2.45 mmol, 1.00 eq.) in methanol (4.90 mL), $NaBH_4$ (370 mg, 9.79 mmol, 4.00 eq.) was added portion-wise, warmed to room temperature and stirred overnight. The reaction mixture was quenched by the addition of water, acidified with HCl (1.00 M) and finally extracted with DCM. The combined organic layers were dried over $MgSO_4$, filtered, and concentrated in vacuo giving rise to (5-(thiophen-2-yl)isoxazol-3-yl)methanol (438 mg, 2.42 mmol) in 99 % yield. A solution of the alcohol (430 mg, 2.37 mmol, 1.00 eq.), Et_3N (0.69 mL, 4.98 mmol, 2.10 eq.) and MsCl (0.24 mL, 3.08 mmol, 1.30 eq.) in DCM (12.00 mL) was stirred for 1 hour at room temperature, quenched with water (100.0 mL) and extracted with DCM. The combined organic layers were dried over $MgSO_4$, filtered, concentrated and immediately used for the next reaction step.

The freshly prepared mesylate (615 mg, 2.37 mmol) and cyclopropylamine (0.33 mL) were dissolved in *i*PrOH (1.0 M), submitted to a microwave device, stirred for 2 h at 100 °C (300 W) and concentrated *in vacuo*. Column chromatography (DCM) rendered **Ox-24** (150 mg, 0.68 mmol) in 29 % yield (over two steps) as yellow solid: ^{1}H-NMR (500 MHz, DMSO-d$_6$): δ_H = 7.79 (dd, $^{3}J_{H,H}$ = 4.9 Hz, $^{4}J_{H,H}$ = 1.2 Hz, 1H), 7.66 (dd, $^{3}J_{H,H}$ = 3.7 Hz, $^{4}J_{H,H}$ = 1.2 Hz, 1H), 7.23 (dd, $^{3}J_{H,H}$ = 4.9 Hz, 3.7 Hz, 1H), 6.80 (s, 1H), 3.77 (s, 2H), 2.85 (br s, 1H), 2.09 (sm, 1H), 0.38 – 0.24 (m, 4H); ^{13}C-NMR (125 MHz, DMSO-d$_6$): δ_C = 164.3, 163.7, 129.0, 128.51, 128.49, 127.4, 99.3, 43.7, 29.7, 6.1; HRMS (ESI, *m/z*): [M+H]$^+$ calculated for $[C_{11}H_{13}N_2OS]^+$: 221.0743, found: 221.0744; combustion analysis (C/H/N/S, *%*): calculated for $[C_{11}H_{12}N_2OS]$ * 0.25 H_2O: 58.77 / 5.60 / 12.46 / 14.26, found: 58.90 / 5.42 / 12.78 / 13.95.

_N_-Cyclopropyl-5-(_p_-tolyl)isoxazol-3-carboxamide (Ox-25)

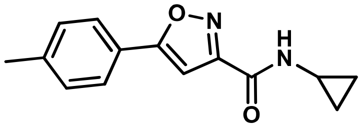

Following general procedure B, step B1 (method B), employing 1-(*p*-tolyl)ethan-1-one (1342 mg, 10.00 mmol) rendered methyl-(*Z*)-2-hydroxy-4-oxo-4-(*p*-tolyl)but-2-enoate (757 mg, 3,43 mmol) in 34 % yield. Reaction of this intermediate (746 mg, 3.39 mmol) according to general procedure B, step B2, gave rise to methyl-5-(*p*-tolyl)isoxazol-3-carboxylate (494 mg, 2.27 mmol) in 67 % yield: ^{1}H-NMR (500 MHz, DMSO-d$_6$): δ_H = 7.84 (d, $^{3}J_{H,H}$ = 8.3 Hz, 2H), 7.41 (s, 1H), 7.36 (d, $^{3}J_{H,H}$ = 8.0 Hz, 2H), 3.92 (s. 3H), 2.37 (s, 3H); ^{13}C-NMR (125 MHz, DMSO-d$_6$): δ_C = 171.3, 159.8, 156.6, 141.0, 129.8, 125.7, 123.4, 100.1, 52.8, 21.0; HRMS (ESI, *m/z*): [M+Na]$^+$ calculated for $[C_{12}H_{11}NO_3Na]^+$: 240.0631, found: 240.0632; combustion analysis (C/H/N, *%*): calculated for $[C_{12}H_{11}NO_3]$: 66.35 / 5.10 / 6.45, found: 66.28 / 5.14 / 6.36; mp: 117 °C.

Employing the corresponding methyl ester (221 mg, 1.00 mmol, 1.00 eq.) and cyclopropylamine (0.14 mL, 2.01 mmol, 2.01 eq.) gave rise to **Ox-25** (125 mg, 0.52 mmol) in 52 % yield: ^{1}H-NMR (500 MHz, DMSO-d$_6$): δ_H = 8.80 (d, $^{3}J_{H,H}$ = 4.3 Hz, 1H), 7.80 (d, $^{3}J_{H,H}$ = 8.3 Hz, 2H), 7.36 (d, $^{3}J_{H,H}$ = 8.0 Hz, 2H), 7.24 (s, 1H), 2.90 – 2.83 (m, 1H), 2.37 (s, 3H), 0.73 – 0.62 (m, 4H); ^{13}C-NMR (125 MHz, DMSO-d$_6$): δ_C = 170.3, 159.7, 159.5, 140.7, 129.8, 125.7, 123.6, 99.1, 22.7, 21.0, 5.6; HRMS (ESI, *m/z*): [M+Na]$^+$ calculated for $[C_{14}H_{14}N_2O_2Na]^+$: 265.0947, found: 265.0948; purity (qNMR, 500 MHz, DMSO-d$_{6.}$ maleic acid): 97 %; mp: 164 °C.

_N_-Cyclopropyl-5-(naphthalen-2-yl)isoxazol-3-carboxamide (Ox-26)

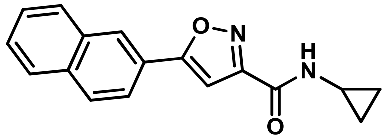

Following general procedure B, step B1 (method B), reaction of 1-(Naphthalen-2-yl)ethan-1-one (1702 mg, 10.00 mmol) provided intermediate methyl-(*Z*)-2-hydroxy-4-(naphthalen-2-yl)-4-oxobut-2-enoate (1722 mg, 6.72 mmol) in 67 % yield. Following general procedure B, step B2, employing methyl-(*Z*)-2-hydroxy-4-(naphthalen-2-yl)-4-oxobut-2-enoate (1711 mg, 6.68 mmol) furnished methyl-5-(naphthalen-2-yl)isoxazol-3-carboxylate (930 mg, 3.67 mmol) in 55 % yield: ^{1}H-NMR (500 MHz, DMSO-d$_6$): δ_H = 8.56 (d, $^{4}J_{H,H}$ = 0.9 Hz, 1H), 8.10 – 7.97 (m, 4H), 7.64 – 7.61 (m, 2H), 7.60 (s, 1H), 3.95 (s, 3H); ^{13}C-NMR (125 MHz, DMSO-d$_6$): δ_C = 171.1, 159.8, 156.7, 133.7, 132.5, 129.0, 128.7, 127.7, 127.1, 125.6, 123.3, 122.7, 101.1, 52.8; HRMS (ESI, *m/z*): [M+Na]$^+$ calculated for $[C_{15}H_{11}NO_3Na]^+$: 276.0631, found:

276.0632; combustion analysis (C/H/N, %): calculated for [$C_{15}H_{11}NO_3$]: 71.14 / 4.38 / 5.53, found: 71.22 / 4.42 / 5.47; mp: 126 °C.

Following general procedure B, step B3, methyl-5-(naphthalen-2-yl)isoxazol-3-carboxylate (380 mg, 1.50 mmol, 1.00 eq) and cyclopropylamine (0.21 mL, 3.02 mmol, 2.01 eq.) were subjected to a microwave device for 4 hours. Column chromatography (DCM) rendered **Ox-26** (142 mg, 0.51 mmol) in 34 % yield: ^{1}H-NMR (500 MHz, DMSO-d$_6$): δ_H = 8.88 (d, $^{3}J_{H,H}$ = 4.6 Hz, 1H), 8.54 (d, $^{4}J_{H,H}$ = 1.2 Hz, 1H), 8.10 – 7.96 (m, 4H), 7.63 – 7.59 (m, 2H), 7.44 (s, 1H), 2.94 – 2.86 (m, 1H), 0.74 – 0.64 (m, 4H); ^{13}C-NMR (125 MHz, DMSO-d$_6$): δ_C = 170.2, 159.7, 159.6, 133.6, 132.6, 129.0, 128.7, 127.6, 127.1, 125.4, 123.6, 122.8, 100.2, 22.8, 5.6; HRMS (ESI, *m/z*): [M+Na]$^+$ calculated for [$C_{17}H_{14}N_2O_2Na$]$^+$: 301.0947, found: 301.0949; combustion analysis (C/H/N, %): calculated for [$C_{17}H_{14}N_2O_2$]: 73.37 / 5.07 / 10.07, found: 73.25 / 5.08 / 9.99; mp: 185 °C.

<u>*N*-Ethyl-*N*-methyl-5-(thiophen-2-yl)isoxazol-3-carboxamide (Ox-27)</u>

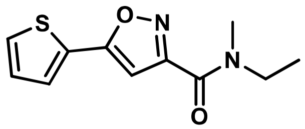

To a solution of **Ox-22** (42 mg, 0.19 mmol) in DMF (1.0 mL), NaH (60 % w/w in mineral oil, 10 mg, 0.40 mmol, 2.10 eq.) and MeI (0.01 mL, 0.20 mmol, 1.05 eq.) were added and stirred for 1 hour at room temperature. The reaction mixture was diluted with DCM, washed with an aqueous solution of LiCl (5 % w/w), dried over $MgSO_4$, filtered, and finally concentrated in vacuo giving rise to **Ox-27** (42 mg, 0.18 mmol) as colorless crystals in 94 % yield: ^{1}H-NMR (500 MHz, DMSO-d$_6$, rotamers): δ_H = 7.86 (dd, $^{3}J_{H,H}$ = 5.0 Hz, $^{4}J_{H,H}$ = 1.0 Hz, 1H, 7.77 (dt, $^{3}J_{H,H}$ = 3.3 Hz, $^{4}J_{H,H}$ = 1.2 Hz, 1H), 7.27 (dd, $^{3}J_{H,H}$ = 5.0 Hz, 3.9 Hz, 1H), 7.09 + 7.08 (s, 3H), 3.50 + 3.44 (q, $^{3}J_{H,H}$ = 7.2 Hz + 7.1 Hz, 2H), 3.08 + 3.00 (s, 3H), 1.17 – 1.12 (m, 3H); ^{13}C-NMR (125 MHz, DMSO-d$_6$, rotamers): δ_C = 164.7, 164.6, 159.9, 159.6, 159.5, 159.3, 130.00, 129.96, 128.63, 128.58, 128.5, 127.50, 127.48, 99.8, 99.6, 45.0, 42.0, 35.5, 32.2, 13.7, 11.7; HRMS (ESI, *m/z*): [M+H]$^+$ calculated for [$C_{11}H_{13}N_2O_2S$]$^+$: 237.0692, found: 237.0693; combustion analysis (C/H/N/S, %): calculated for [$C_{11}H_{12}N_2O_2S$]: 55.92 / 5.12 / 11.86 / 13.57, found: 55.60 / 4.96 / 11.86 / 13.21; mp: 82 °C.

<u>*N*-Cyclopropyl-3-(thiophen-2-yl)-1,2,4-oxadiazole-5-carboxamide (Ox-28)</u>

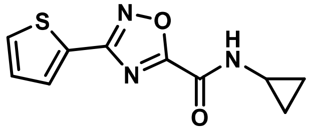

To a solution of thiophene-2-carbonitril in EtOH (1.00 mL, 10.72 mmol), NH_2OH^*HCl (1.50 eq.) and DIPEA (1.60 eq.) were added. The reaction mixture was heated to reflux for 4 h, concentrated *in vacuo*, redissolved in EtOAc/H_2O and extracted with EtOAc. The combined organic phase was washed with H_2O (x2) and subsequently with a saturated aqueous solution of NaCl, dried over $MgSO_4$ and concentrated *in vacuo* to afford *N*-hydroxythiophene-2-carboximidamide as a colourless solid (1506 mg, 10.59 mmol) in 99 % yield: ^{1}H-NMR (300 MHz, DMSO-d$_6$): δ_H = 9.59 (s, 1H), 7.50 – 7.34 (m, 2H), 7.04 (dd, $^3J_{H,H}$ = 5.1, 3.6 Hz, 1H, 5.90 (br s, 2H). [3]
In the following step, *N*-hydroxythiophene-2-carboximidamide (284 mg, 2.00 mmol, 1.00 eq.), methyl 2-chloro-2-oxoacetate (0.55 mL, 6.00 mmol, 3.00 eq.) and K_2CO_3 (2.00 eq.) were suspended in MeCN (0.5 M) under an argon-atmosphere, submitted to a microwave device and stirred for 1 h at 120 °C (150 W). The reaction mixture was filtered, concentrated under reduced pressure and finally extracted with EtOAc. The combined organic phase was washed with a saturated aqueous solution of NaCl, dried over $MgSO_4$ and concentrated *in vacuo*. Column chromatography (dichloromethane) gave rise to methyl 3-(thiophen-2-yl)-1,2,4-oxadiazole-5-carboxylate (231 mg, 1.10 mmol) as light-yellow solid in 55 % yield: ^{1}H-NMR (500 MHz, DMSO-d$_6$): δ_H = 7.95 (dd, $^3J_{H,H}$ = 5.0 Hz, $^4J_{H,H}$ = 1.3 Hz, 1H), 7.89 (dd, $^3J_{H,H}$ = 3.7 Hz, $^4J_{H,H}$ = 1.1 Hz, 1H), 7.30 (dd, $^3J_{H,H}$ = 4.8, 3.7 Hz, 1H), 3.99 (s, 3H); ^{13}C-NMR (125 MHz, DMSO-d$_6$): 166.6, 164.6, 154.0, 131.5, 130.7, 128.7, 126.3, 53.8; HRMS (ESI, *m/z*): [M+Na]$^+$ calculated for $[C_8H_6N_2O_3NaS]^+$: 232.9991, found: 232.9992; combustion analysis (C/H/N/S, *%*): calculated for $[C_8H_6N_2O_3S]$: 45.71 / 2.88 / 13.33 / 15.25, found: 45.74 / 2.90 / 13.32 / 15.17; mp: 75 °C. Following general procedure B, step B3 reaction of the methyl ester (158 mg, 0.75 mmol, 1.00 eq) and cyclopropylamine (0.10 mL, 1.50 mmol, 2 eq.) rendered **Ox-28** (143 mg, 0.61 mmol) as colourless crystals in 81 % yield: ^{1}H-NMR (500 MHz, DMSO-d$_6$): δ_H = 9.49 (s, 1H), 7.93 (dd, $^3J_{H,H}$ = 5.0 Hz, $^4J_{H,H}$ = 1.3 Hz, 1H), 7.86 (dd, $^3J_{H,H}$ = 3.7 Hz, $^4J_{H,H}$ = 1.2 Hz, 1H), 7.30 (dd, $^3J_{H,H}$ = 5.2, 3.7 Hz, 1H), 2.94 – 2.89 (m, 1H), 0.77 – 0.70 (m, 4H); ^{13}C-NMR (125 MHz, DMSO-d$_6$): 169.0, 164.1, 153.8, 131.3, 130.5, 128.6, 126.6, 23.1, 5.5; HRMS (ESI, *m/z*): [M+Na]$^+$ calculated for $[C_{10}H_9N_3O_2NaS]$: 258.0308, found: 258.0308; combustion analysis (C/H/N/S, *%*): calculated for $[C_{10}H_9N_3O_2S]$: 51.05 / 3.86 / 17.86 / 13.63, found: 51.06 / 3.87 / 17.83 / 13.34.; mp: 125 °C.

5-(4-(Benzyloxy)phenyl)-*N*-cyclopropylisoxazol-3-carboxamide (Ox-29)

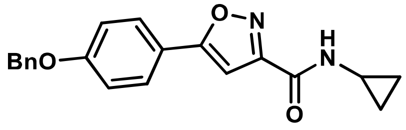

Following general procedure B step B1 (method B) reaction of 1-(4-(benzyloxy)phenyl)ethan-1-one (905 mg, 4.00 mmol) provided intermediate methyl-(*Z*)-4-(4-(benzyloxy)phenyl)-2-hydroxy-4-oxobut-

2-enoate (433 mg, 1.39 mmol) in 35 % yield. Reaction of this intermediate (433 mg, 1.39 mmol) following general procedure B, step B2, gave rise to methyl-5-(4-(benzyloxy)phenyl)isoxazol-3-carboxylate (238 mg, 0.77 mmol) in 55 % yield: ^{1}H-NMR (500 MHz, DMSO-d$_6$): δ_H = 7.89 (d, $^3J_{H,H}$ = 8.9 Hz, 2H), 7.50 – 7.31 (m, 6H), 7.18 (d, $^3J_{H,H}$ = 8.9 Hz, 2H), 5.20 (s, 2H), 3.92 (s, 3H); ^{13}C-NMR (125 MHz, DMSO-d$_6$): δ_C = 171.1, 160.3, 159.9, 156.5, 136.5, 128.4, 127.9, 127.7, 127.6, 118.9, 115.6, 99.3, 69.4, 52.7; HRMS (ESI, *m/z*): [M+H]$^+$ calculated for [$C_{18}H_{16}NO_4$]$^+$: 310.1074, found: 310.1079; combustion analysis (C/H/N, *%*): calculated for [$C_{18}H_{15}NO_4$]: 69.89 / 4.89 / 4.53, found: 69.92 / 4.89 / 4.42; mp: 139 °C.

Following general procedure B, step B3, methyl-5-(4-(benzyloxy)phenyl)isoxazol-3-carboxylate (302 mg, 0.98 mmol, 1.00 eq.) and cyclopropylamine (0.14 mL, 1.95 mmol, 2.00 eq.) were subjected to a microwave device (300W) for 4 hours. Column chromatography (DCM) rendered **Ox-29** (253 mg, 0.76 mmol) in 77 % yield: ^{1}H-NMR (500 MHz, DMSO-d$_6$): δ_H = 8.78 (d, $^3J_{H,H}$ = 4.6 Hz, 1H), 7.85 (d, $^3J_{H,H}$ = 8.9 Hz, 2H), 7.49 – 7.31 (m, 5H), 7.19 – 7.15 (m, 3H), 5.20 (s, 2H), 2.89 – 2.84 (m, 1H), 0.74 – 0.61 (m, 4H); ^{13}C-NMR (125 MHz, DMSO-d$_6$): δ_C = 170.2, 160.2, 159.7, 159.5, 136.6, 128.4, 127.9, 127.7, 127.5, 119.2, 115.5, 98.3, 69.4, 22.7, 5.6; HRMS (ESI, *m/z*): [M+Na]$^+$ calculated for [$C_{20}H_{18}N_2O_3Na$]$^+$: 357.1210, found: 357.1209; combustion analysis (C/H/N, *%*): calculated for [$C_{20}H_{18}N_2O_3$]: 71.84 / 5.43 / 8.38, found: 71.58 / 5.44 / 8.32; mp: 180 °C.

N-Cyclopropyl-5-(4-hydroxyphenyl)isoxazole-3-carboxamide (Ox-31)

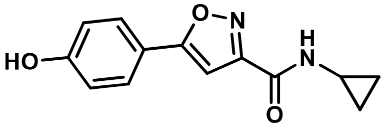

1-(4-Hydroxyphenyl)ethan-1-one (1362 mg, 10.00 mmol, 1.00 eq.), imidazole (1021 mg, 15.00 mmol, 1.50 eq.) and TBSCl (1959 mg, 13.00 mmol, 1.30 eq.) were dissolved in DMF (17.00 mL) and the resulting solution was stirred overnight at room temperature. The reaction mixture was diluted with *c*Hex / Et_2O (1:1, 100 mL), the organic layer washed with water H_2O (3x), a saturated aqueous NaCl solution, dried over $MgSO_4$, and concentrated in vacuo yielding 1-(4-((*tert*-butyldimethylsilyl)oxy)phenyl)ethan-1-one (2410 mg, 9.62 mmol) as colorless solid in 96 % yield. [4]

According to general procedure B step B1 (A2) reaction of 1-(4-((*tert*-butyldimethylsilyl)oxy)phenyl)ethan-1-on (2300 mg, 9.18 mmol) rendered methyl-(*Z*)-4-(4-((tert-butyldimethylsilyl)oxy)phenyl)-2-hydroxy-4-oxobut-2-enoate (1362 mg, 4.05 mmol) in 44 % yield.

Reaction of methyl-(*Z*)-4-(4-((tert-butyldimethylsilyl)oxy)phenyl)-2-hydroxy-4-oxobut-2-enoate (1362 mg, 4.05 mmol) and NH_2OH^*HCl (general procedure B step B2) furnished methyl-5-(4-hydroxyphenyl)isoxazol-3-carboxylate as purple solid (52 % yield, crude product) which was used

without further purification in the next step. Utilization of the crude reaction product and cyclopropylamine (0.14 mL) according to procedure B, step B3 gave rise to *N*-cyclopropyl-5-(4-hydroxyphenyl)isoxazole-3-carboxamide **Ox-31** (122 mg, 0.50 mmol) in 50 % yield: ^{1}H-NMR (500 MHz, DMSO-d$_6$): δ_H = 10.09 (s, 1H), 8.76 (d, $^{3}J_{H,H}$ = 4.3 Hz, 1H), 7.73 (d, $^{3}J_{H,H}$ = 8.9 Hz, 2H), 7.06 (s, 1H), 6.90 (d, $^{3}J_{H,H}$ = 8.9 Hz, 2H), 2.86 (sm, 1H), 0.74 – 0.58 (m, 4H); ^{13}C-NMR (125 MHz, DMSO-d$_6$): δ_C = 170.6, 159.8, 159.7, 159.4, 127.6, 117.5, 116.0, 97.5, 22.7, 5.6; HRMS (ESI, *m/z*): [M+Na]$^+$ calculated for $[C_{13}H_{12}N_2O_3Na]^+$: 267.0740, found: 267.0740; combustion analysis (C/H/N, *%*): calculated for $[C_{13}H_{12}N_2O_3]$: 63.93 / 4.95 / 11.47, found: 63.89 / 4.98 / 11.42; mp: 191 °C.

<u>*N*-Cyclopropyl-5-(4-(hydroxymethyl)phenyl)isoxazol-3-carboxamide (</u>Ox-32)

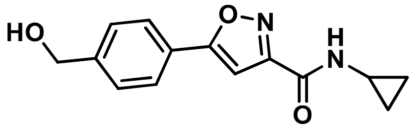

1-(4-(Hydroxymethyl)phenyl)ethan-1-one (6758 mg, 45.00 mmol, 1.00 eq.), Et_3N (9.40 mL, 67.50 mmol, 1.50 eq.), DMAP (catalytic amount) and TBSCl (8817 mg, 58.50 mmol, 1.30 eq.) were dissolved in DCM (90.00 mL) and stirred at room temperature overnight. The solvent was removed in vacuo, the remaining residue re-dissolved in *c*Hex, stirred for 3 hours and finally filtered to remove insoluble material. The remaining organic phase was washed with water, a saturated aqueous NaCl solution, dried over $MgSO_4$, filtered, and concentrated in vacuo. Bulb to bulb distillation gave rise to 1-(4-(((tert-butyldimethylsilyl)oxy)methyl)phenyl)ethan-1-one (11.71 g, 44.28 mmol) as slightly yellow oil in 98 % yield.

Following general procedure B, step B1 (method A2), reaction of 1-(4-(((tert-butyldimethylsilyl)oxy)-methyl)phenyl)ethan-1-one (2300 mg, 8.69 mmol) provided intermediate methyl-(Z)-4-(4-(((tert-butyldimethylsilyl)oxy)methyl)phenyl)-2-hydroxy-4-oxobut-2-enoate (1613 mg, 4.60 mmol) in 53 % yield.

Reaction of this intermediate (1613 mg, 4.60 mmol) following general procedure B step B2 gave rise to methyl-5-(4-hydroxyphenyl)isoxazol-3-carboxylate (414 mg, 1.60 mmol) in 39 % yield: ^{1}H-NMR (500 MHz, DMSO-d$_6$): δ_H = 7.91 (d, $^{3}J_{H,H}$ = 8.6 Hz, 2H), 7.49 (d, $^{3}J_{H,H}$ = 8.6 Hz, 2H), 7.45 (s, 1H), 5.33 (t, $^{3}J_{H,H}$ = 5.7 Hz, 1H), 4.57 (d, $^{3}J_{H,H}$ = 5.7 Hz, 2H), 3.93 (s, 3H); ^{13}C-NMR (125 MHz, DMSO-d$_6$): δ_C = 171.2, 159.8, 156.6, 145.8, 127.0, 125.6, 124.4, 100.3, 62.4, 52.8; HRMS (ESI, *m/z*): [M+Na]$^+$ calculated for $[C_{12}H_{11}NO_4Na]^+$: 256.0580, found: 256.0584; combustion analysis (C/H/N, *%*): calculated for $[C_{12}H_{11}NO_4]$: 61.80 / 4.75 / 6.01, found: 61.31 / 4.76 / 5.85; mp: 131 °C.

Following general procedure B, step B3, methyl-5-(4-hydroxyphenyl)isoxazol-3-carboxylate (233 mg, 1.00 mmol, 1.00 eq.) and cyclopropylamine (0.14 mL, 2.00 mmol, 2.00 eq.) rendered **Ox-32** (147 mg,

0.57 mmol) in 57 % yield: ^{1}H-NMR (500 MHz, DMSO-d$_6$): δ_H = 8.79 (d, $^3J_{H,H}$ = 4.2 Hz, 1H), 7.88 (d, $^3J_{H,H}$ = 8.3 Hz, 2H), 7.48 (d, $^3J_{H,H}$ = 8.3 Hz, 2H), 7.28 (s, 1H), 5.34 (t, $^3J_{H,H}$ = 5.9 Hz, 1H), 4.57 (d, $^3J_{H,H}$ = 5.7 Hz, 2H), 2.87 (sm, 1H), 0.80 – 0.55 (m, 4H); ^{13}C-NMR (125 MHz, DMSO-d$_6$): δ_C = 170.3, 159.6, 159.5, 145.6, 127.0, 125.5, 124.7, 99.3, 62.4, 22.7, 5.6; HRMS (ESI, *m/z*): [M+Na]$^+$ calculated for [$C_{14}H_{14}N_2O_3Na$]$^+$: 281.0897, found: 281.0900; combustion analysis (C/H/N, *%*): calculated for [$C_{14}H_{14}N_2O_3$]: 65.11 / 5.46 / 10.85, found: 65.16 / 5.50 / 10.75; mp: 179 °C.

***N*-Cyclopropyl-5-(*o*-tolyl)isoxazol-3-carboxamide (Ox-36)**

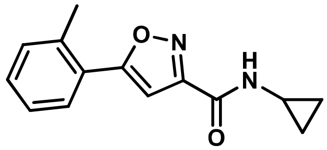

Following general procedure B, step B1, reaction of 1-(*o*-tolyl)ethan-1-one (2013 mg, 15.00 mmol) provided intermediate methyl-(*Z*)-2-hydroxy-4-oxo-4-(*o*-tolyl)but-2-enoate (1122 mg, 5.10 mmol) in 34 % yield.

Following general procedure B, step B2, employing methyl-(*Z*)-2-hydroxy-4-oxo-4-(*o*-tolyl)but-2-enoate (1122 mg, 5.10 mmol) rendered methyl-5-(*o*-tolyl)isoxazol-3-carboxylate (737 mg, 3.39 mmol) in 66 % yield: ^{1}H-NMR (500 MHz, DMSO-d$_6$): δ_H = 7.77 (dd, $^3J_{H,H}$ = 7.7 Hz , $^4J_{H,H}$ = 1.4 Hz, 1H), 7.49 - 7.34 (m, 3H), 7.21 (s, 1H), 3.94 (s, 3H), 2.48 (s, 3H); ^{13}C-NMR (125 MHz, DMSO-d$_6$): δ_C = 171.1, 159.8, 156.2, 136.1, 131.4, 130.7, 128.4, 126.4, 125.6, 103.3, 52.8, 20.7; HRMS (ESI, *m/z*): [M+Na]$^+$ calculated for [$C_{12}H_{11}NO_3Na$]$^+$: 240.0631, found: 240.0633; combustion analysis (C/H/N, *%*): calculated for [$C_{12}H_{11}NO_3$]: 66.35 / 5.10 / 6.45, found: 66.28 / 5.14 / 6.41; mp: 51 °C.

Reaction of methyl-5-(*o*-tolyl)isoxazol-3-carboxylate (348 mg, 1.6 mmol, 1.00 eq.) and cyclopropylamine (0.22 mL, 3.16 mmol, 1.98 eq.) following general procedure B, step B3, rendered **Ox-36** (97 mg, 0.40 mmol) after column chromatography (*c*Hex/EtOAc, 4:1) in 25 % yield: ^{1}H-NMR (500 MHz, DMSO-d$_6$): δ_H = 8.83 (d, $^3J_{H,H}$ = 4.3 Hz, 1H), 7.72 (dd, $^3J_{H,H}$ = 7.6 Hz, $^4J_{H,H}$ = 1.3 Hz, 1H), 7.45 - 7.35 (m, 3H), 7.07 (s, 1H), 2.87 (sm, 1H), 2.47 (s, 3H), 0.73 - 0.61 (m, 4H); ^{13}C-NMR (125 MHz, DMSO-d$_6$): δ_C = 170.1, 159.6, 159.1, 135.8, 131.8, 130.2, 128.0, 126.4, 125.9, 102.3, 22.7, 20.8, 5.6; HRMS (ESI, *m/z*): [M+Na]$^+$ calculated for [$C_{14}H_{14}N_2O_2Na$]$^+$: 265.0947, found: 265.0949; combustion analysis (C/H/N, *%*): calculated for [$C_{14}H_{14}N_2O_2$]: 69.41 / 5.82 / 11.56, found: 69.22 / 5.84 / 11.49; mp: 105 °C.

***N*-Cyclopropyl-5-(*m*-tolyl)isoxazol-3-carboxamide (Ox-37)**

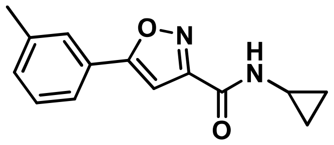

Reaction of 1-(*m*-tolyl)ethan-1-one (2012 mg, 15.00 mmol) following general procedure B, step B1 (method A) furnished methyl-(*Z*)-2-hydroxy-4-oxo-4-(*m*-tolyl)but-2-enoate (1120 mg, 5.09 mmol) in 34 % yield.

Subsequent reaction of this intermediate 1120 mg, 5.09 mmol) according to general procedure B, step B2 gave rise to methyl-5-(*m*-tolyl)isoxazol-3-carboxylate (746 mg, 3.34 mmol) in 67 % yield: ^{1}H-NMR (500 MHz, DMSO-d$_6$): δ_H = 7.79 (s, 1H), 7.74 (d, $^3J_{H,H}$ = 7.7 Hz, 1H), 7.45 (s, 1H), 7.44 (d, $^3J_{H,H}$ = 7.7 Hz, 1H), 7.36 (d, $^3J_{H,H}$ = 7.7 Hz, 1H), 3.93 (s, 3H), 2.39 (s, 3H); ^{13}C-NMR (125 MHz, DMSO-d$_6$): δ_C = 171.2, 159.8, 156.6, 138.7, 131.6, 129.1, 126.1, 125.9, 122.9, 100.6, 52.7, 20.8; HRMS (ESI, *m/z*): [M+Na]$^+$ calculated for [$C_{12}H_{11}NO_3Na$]$^+$: 240.0631, found: 240.0634; mp: 118 °C.

Reaction of methyl-5-(*m*-tolyl)isoxazol-3-carboxylate (326 mg, 1.50 mmol, 1.00 eq.) and cyclopropylamine (0.20 mL, 2.87 mmol, 1.91 eq.) following general procedure B, step B3, rendered **Ox-37** (66 mg, 0.27 mmol) after column chromatography (*c*Hex/EtOAc, 4:1) in 18 % yield: ^{1}H-NMR (500 MHz, DMSO-d$_6$): δ_H = 8.80 (d, $^3J_{H,H}$ = 4.3 Hz, 1H), 7.77 - 7.72 (m, 1H), 7.72 - 7.68 (m, 1H), 7.43 (t, $^3J_{H,H}$ = 7.7 Hz, 1H), 7.36 - 7.32 (m, 1H), 7.24 (s, 1H), 2.90 – 2.83 (m, 1H), 2.37 (s, 3H), 0.73 – 0.62 (m, 4H); ^{13}C-NMR (125 MHz, DMSO-d$_6$): δ_C = 170.3, 159.6, 159.5, 138.7, 131.4, 129.1, 126.2, 126.1, 122.9, 99.6, 22.7, 20.8, 5.5; HRMS (ESI, *m/z*): [M+Na]$^+$ calculated for [$C_{14}H_{14}N_2O_2Na$]$^+$: 265.0947, found: 265.0951; combustion analysis (C/H/N, *%*): calculated for [$C_{14}H_{14}N_2O_2$]: 69.41 / 5.82 / 11.56, found: 69.14 / 5.82 / 11.52; mp: 107 °C.

5-Cyclohexyl-*N*-cyclopropylisoxazole-3-carboxamide (Ox-38)

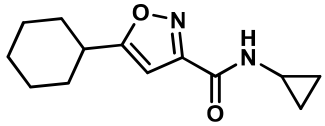

According to general procedure B, step B1 (method B), utilization of 1-cyclohexylethan-1-one (1893 mg, 15.00 mmol) afforded methyl (*Z*)-4-cyclohexyl-2-hydroxy-4-oxobut-2-enoate (1715 mg, 8.07 mmol) in 54 % yield. Reaction of this intermediate (1715 mg, 8.07 mmol) following general procedure B, step B2, gave rise to methyl 5-cyclohexylisoxazole-3-carboxylate (328 mg, 1.57mmol) in 19 % yield: ^{1}H-NMR (500 MHz, DMSO-d$_6$): δ_H = 6.65 (s, 1H), 3.88 (s, 3H), 2.89 (m, 1H), 2.00 – 1.20 (m, 10H); ^{13}C-NMR (125 MHz, DMSO-d$_6$) δ_C = 179.5, 160.0, 155.6, 100.1, 52.6, 35.4, 30.3, 25.1, 24.9; HRMS (ESI, *m/z*): [M+H]$^+$ calculated for [$C_{11}H_{16}NO_3$]: 210.1152, found: 210.1128; combustion analysis (C/H/N, *%*): calculated for [$C_{11}H_{15}NO_3$]: 63.14 / 7.23 / 6.69, found: 62.96 / 7.17 / 6.56; mp: 71 °C.

Reaction of the methyl ester (300 mg, 1.43 mmol, 1.00 eq.) with cyclopropylamine (164 mg, 2.87 mmol, 2.0 eq.) according to general procedure B, step B3, followed by column chromatography (*c*Hex / EtOAc, 4:1) provided **Ox-38** (60 mg, 0.26 mmol) in 18 % yield as colorless crystals: ^{1}H-NMR (500 MHz, DMSO-d_6): δ_H = 8.68 (d, $^{3}J_{H,H}$ = 4.3 Hz, 1H), 6.50 (s, 1H), 2.88 – 2.80 (m, 2H), 2.01 – 1.20 (m, 10H), 0.70 – 0.57 (m, 4H); ^{13}C-NMR (125 MHz, DMSO-d_6) δ_C = 178.4, 159.9, 158.6, 98.9, 35.3, 30.5, 25.2, 24.9, 22.6, 5.5; HRMS (ESI, *m/z*): $[M+Na]^+$ calculated for $[C_{13}H_{18}N_2O_2Na]$: 257.1260; found: 257.1264; combustion analysis (C/H/N, *%*): calculated for $[C_{13}H_{18}N_2O_2]$: 66.64 / 7.74 / 11.96, found: 66.62 / 7.69 / 11.93; mp: 139 °C.

Cited literature

[1] J. Schneider, J. Hsieh, D. Frantz, S.L. Mcknight, J.M. Ready, U.S. (2012), US 8193225 B2

[2] J. L. Russell, S. C. Goetsch, H. R. Aguilar, H. Coe, X. Luo, N. Liu, E. van Rooij, D. E. Frantz, J. W. Schneider, *ACS Chem. Biol.* **2012**, *7*, 1077–1083.

[3] M. Flipo, M. Desroses, N. Lecat-Guillet, B. Dirié, X. Carette, F. Leroux, C. Piveteau, F. Demirkaya, Z. Lens, P. Rucktooa, et al., *J. Med. Chem.* **2011**, *54*, 2994–3010.

[4] Y. T. Han, K. Kim, G.-I. Choi, H. An, D. Son, H. Kim, H.-J. Ha, J.-H. Son, S.-J. Chung, H.-J. Park, et al., *Eur. J. Med. Chem.* **2014**, *79*, 128–142.

Table S1:

Hh reporter luciferase screen (8xGliBS-Luc) in MCF7:

	Compound	Concentration in assay	Description	mean RLU [%] **(normalized to total protein and rel. to DMSO)**	**SEM**
	**DMSO**	0.5%	Solvent	100	0
**HAT-Inh**	**C646**	10 µM	An inhibitor of the HAT p300	96,31690053	11,8129211
	**3-amino Benzamide**	10 µM	Inhibitor of poly(ADP-ribose) polymerases	81,42333017	23,24565018
	**CAY10669**	10 µM	An inhibitor of PCAF	76,00869723	26,65092399
	**Delphinidin (chloride)**	10 µM	Natural vasorelaxant and inhibitor of EGFRs and HATs	78,12342577	22,19011266
	**CPTH2 (hydrochloride)**	10 µM	A HAT inhibitor	111,3996295	36,72324155
	**Anacardic Acid**	10 µM	A histone acetyltransferase inhibitor	114,9807391	24,6367256
	**I-CBP112 (hydrochloride)**	10 µM	A selective inhibitor of CBP and EP300	131,3945123	36,81326003
**SIRT-Inh**	**Sirtinol**	10 µM	Inhibitor of sirtuin deacetylases	114,0608629	43,95775591
	**JGB1741**	10 µM	A SIRT1 inhibitor	95,29954353	38,07229752
	**AGK2**	10 µM	Inhibitor of SIRT2	62,658506	12,01734789
	**Salermide**	10 µM	A sirtuin inhibitor	130,338155	36,62744695
	**SIRT1/2 Inhibitor IV**	10 µM	Cell-permeable inhibitor of SIRT1 and SIRT2	124,2546724	22,33046412
	**CAY10591**	10 µM	A SIRT1 activator	88,19312997	18,42930001
	**(±)-EX-527**	10 µM	A SIRT1 inhibitor	113,3551543	27,70540382
	**Nicotinamide**	10 µM	An amide form of vitamin B3 (niacin)	91,50706153	6,984817422
**PAD-Inh**	**Cl-Amidine (hydrochloride)**	10 µM	A PAD inhibitor	139,4459063	14,48005493
	**F-Amidine (trifluoroacetate salt)**	10 µM	A PAD inhibitor	105,8232819	21,95641831
**HDAC-Inh**	**CAY10398**	10 µM	A potent, cost-effective histone deacetylase inhibitor	152,3711743	70,77881585
	**Trichostatin A**	10 µM	A potent, reversible inhibitor of histone deacetylases	92,91662037	38,23185572
	**Splitomicin**	10 µM	Inhibitor of yeast Sir2p	91,52741293	8,00740162
	**Chidamide**	10 µM	An HDAC inhibitor	311,5718675	62,3104075
	**Pyroxamide**	10 µM	A HDAC inhibitor	108,7175481	32,68024592
	**SAHA**	10 µM	An HDAC inhibitor	130,185456	43,43315565
	**KD 5170**	10 µM	An inhibitor of class I and II HDACs	241,3853537	64,74552215
	**BML-210**	10 µM	A potent, synthetic HDAC inhibitor	166,1872597	28,91335596
	**ACY-1215**	10 µM	HDAC6 Inh	146,378209	31,13839157
	**SB939**	10 µM	A pan-HDAC inhibitor	82,3576149	20,58908315
	**PCI 34051**	10 µM	A potent, selective HDAC8 inhibitor	189,7479261	63,66145656
	**4-iodo-SAHA**	10 µM	A potent SAHA analog	220,3846463	65,83006693
	**Scriptaid**	10 µM	HDAC inhibitor	266,6133442	182,2241748
	**Suberohydroxamic Acid**	10 µM	HDAC inhibitor	80,38619547	19,51987767
	**Apicidin**	10 µM	A cell-permeable HDAC inhibitor	44,2445875	8,092029925
	**ITF 2357**	10 µM	HDAC inhibitor with anti-inflammatory and antineoplastic activities	85,5265122	55,86186546
	**Valproic Acid (sodium salt)**	10 µM	An analog of valeric acid	103,6173897	44,19445851
	**Sodium Butyrate**	10 µM	A short-chain fatty acid and HDAC inhibitor	123,2765742	41,40049694
	**CAY10603**	10 µM	An exceptionally potent inhibitor of HDAC6	130,8464154	29,89002245
	**CBHA**	10 µM	Inhibitor of histone deacetylases	200,393551	60,19494243
	**M 344**	10 µM	HDAC inhibitor	230,6836607	79,56793923
	**Oxamflatin**	10 µM	Inhibitor of histone deacetylases	184,573451	72,17046632
	**Pimelic Diphenylamide 106**	10 µM	A tight-binding inhibitor of class I HDACs	285,000631	15,00896
	**(S)-HDAC-42**	10 µM	Inhibitor of HDACs	126,6023896	61,39795726
	**MS-275**	10 µM	A histone deacetylase inhibitor	280,489598	117,765188
	**HNHA**	10 µM	HDAC inhibitor	97,13139027	8,192142564
**HMT-Inh**	**UNC0321 (trifluoroacetate salt)**	10 µM	A highly potent inhibitor of G9a histone methyltransferase	85,030968	39,62653339
	**UNC0638**	10 µM	A G9a and GLP histone methyltransferase inhibitor	113,9436464	36,20878987
	**Zebularine**	10 µM	A DNA methyltransferase inhibitor	75,69334617	23,15015999
	**(-)-Neplanocin A**	10 µM	Irreversible SAH hydrolase inhibitor	142,1967218	43,80896699
	**5-Azacytidine**	10 µM	A DNA methyltransferase inhibitor	116,9117598	55,05301799
	**MI-2 (hydrochloride)**	10 µM	An inhibitor of menin-MLL interactions	187,5501167	101,9586034
	**MI-nc (hydrochloride)**	10 µM	A negative control for MI-2	133,8774903	19,0638454
	**Chaetocin**	10 µM	Inhibitor of lys9-specific HMTs	28,5188832	20,33329324
	**WDR5-0103**	10 µM	An inhibitor of WDR5 peptide binding	77,01088387	14,84731178
	**UNC0224**	10 µM	A potent inhibitor of G9a histone methyltransferase	184,9552968	45,5349343
	**S-Adenosylhomocysteine**	10 µM	Amino Acid Derivative	165,7071664	46,54281155
	**BIX01294 (hydrochloride hydrate)**	10 µM	An inhibitor of G9a histone methyltransferase	53,0042893	18,35956451
**BET-Inh**	**PFI-1**	10 µM	A BET bromodomain inhibitor	84,8992166	28,64033514
	**(+)-JQ1**	10 µM	A selective inhibitor of BET bromodomains	31,09761635	1,49725865
	**(−)-JQ1**	10 µM	The inactive stereoisomer of a BET bromodomain inhibitor	153,7976189	58,78773208
	**PFI-3**	10 µM	Probe for bromodomains of SMARCA2/4 and PB1(bromodomain 5)	121,694926	13,6298604
**DNMT-Inh**	**Decitabine**	10 µM	A 2’ deoxy analog of 5-azacytidine	163,6473651	96,10772273
	**Gemcitabine**	10 µM	An anticancer nucleoside analog; DNA-MT-Inhib.	80,72765833	24,94488602
	**Lomeguatrib**	10 µM	Inactivator of O6-methylguanine-DNA methyltransferase	142,2533415	56,26816868
	**RG-108**	10 µM	DNA methyltransferase inhibitor	93,21509277	7,506607186
	**2',3',5'-triacetyl-5-Azacytidine**	10 µM	A prodrug form of 5-azacytidine	119,2624627	21,94341168
	**Sinefungin**	10 µM	A methyltransferase inhibitor	127,3761643	27,82762212
**HDM-Inh**	**IOX1**	10 µM	A 2-oxoglutarate oxygenase inhibitor	70,62149707	19,44142348
	**Octyl-α-ketoglutarate**	10 µM	A cell-permeable form of α-ketoglutarate	158,7908981	79,32505703
	**Daminozide**	10 µM	Selective inhibitor of KDM2/7 histone demethylases	91,1544438	22,22891709
	**GSK-J1 (sodium salt)**	10 µM	A dual inhibitor of JMJD3 and UTX	206,7227938	121,2236308
	**GSK-J2 (sodium salt)**	10 µM	A negative control compound for GSK-J1	117,3448177	58,0016317
	**GSK-J4 (hydrochloride)**	10 µM	Prodrug of a selective H3K27 histone demethylase inhibitor	54,83999017	8,046000051
	**GSK-J5 (hydrochloride)**	10 µM	A negative control compound for GSK-J4	78,71332593	25,65397294
	**3-Deazaneplanocin A**	10 µM	An inhibitor of lysine methyltransferase EZH2	198,4617523	46,32249397
	**GSK343**	10 µM	A selective, cell-permeable EZH2 inhibitor	171,5361917	29,94173021
	**UNC1999**	10 µM	A selective, cell-permeable EZH2 inhibitor	121,4363806	22,78631728
	**JIB04**	10 µM	Jumonji-Inh	138,885139	11,38483711
	**Tranylcypromine (hydrochloride)**	10 µM	An irreversible, mechanism-based inhibitor of LSD1	87,03578493	1,80770441
	**N-Oxalylglycine**	10 µM	An inhibitor of JMJD2 histone demethylases	130,0935957	50,59828383
**Other**	**Ellagic Acid**	10 µM	Polyphenolic antioxidant, DNA-Topoisomeraase-Inh	116,8468078	30,6209454
	**Phthalazinone pyrazole**	10 µM	Potent, selective inhibitor of Aurora kinase A	90,59568817	30,36985365
	**CCG-100602**	10 µM	A Rho/MKL1 transcriptional pathway inhibitor	131,9870906	40,81779615
	**Isoliquiritigenin**	10 µM	A flavonoid with diverse biological activities	223,5112213	136,7402244
	**BSI-201**	10 µM	A PARP1 inhibitor	133,7598507	80,81793712
	**1-Naphthoic Acid**	10 µM	A synthetic intermediate	62,12763017	40,79193638
	**Sodium 4-Phenylbutyrate**	10 µM	A chemical chaperone	57,06994597	12,69327374
	**Tenovin-1**	10 µM	A small molecule activator of p53	86,64301937	16,38219028
	**Tenovin-6**	10 µM	A small molecule activator of p53	47,53935297	15,76489771
	**Nutlin3**	10 µM	A small molecule activator of p53, p53/MDM2-inh.	111,0214641	42,95990128
	**SAG**	100 nM	SMO agonist	125,5487825	5,7361305
	**Quercetin**	2 mM	pleiotropic Flavonoid	171,0033617	81,29308191
	**PFL_64176**	10 µM	Activator of Calcium Channels	202,9814877	81,29288709
	**PF-573228**	10 µM	FAK-Inh	75,61167107	10,53614627
	**AY9944**	10 µM	DHCR7-Inh (Cholesterol Biosynthesis)	89,55044277	15,45028525
	**Kenpaullone**	10 µM	CDK-Inh	64,6525251	28,59356294
	**Parvalanol A**	10 µM	CDK-Inh	93,09543877	8,375583431
	**ISX**	10 µM	unknown	878,5463333	88,4873115
	**Mirin**	10 µM	Inhibitor of the DNA damage sensor MRN	111,5183636	33,71259831
	**AMI-1 (sodium salt)**	10 µM	A cell permeable inhibitor of PRMTs	90,44831567	18,30448535
	**UNC1215**	10 µM	Potent L3MBTL3 domain inhibitor	93,7972943	21,13068158

trans-Resveratrol	10 µM	A polyphenolic phytoalexin	211,611419	46,87983152
2,4-DPD	10 µM	A cell permeable, competitive inhibitor of HIF-PH	69,33914923	3,606864203
DMOG	10 µM	HIF-1α prolyl hydroxylase inhibitor	80,0091028	17,14436966
Piceatannol	10 µM	A potent resveratrol analog	218,3660953	56,33310183
SB203580	10 µM	p38-MAPK14 Inh	143,9894313	24,86053685
Nifedipine	10 µM	Calcium Antagonist	128,5830047	8,043541536
BAPTA	10 µM	Calcium Chelator	177,750381	38,74036959
RSC-133	10 µM	Induces pluripotency in somatic cells	105,5669004	52,77707414
KN93	10 µM	CaMK-Inh	104,995363	5,070804238
MK801	10 µM	NMDAR-Antagonist	194,8718307	74,28277805
Bortezomib	1 µM	Proteasome Inh	29,34833413	4,032231046
Suramin (sodium salt)	10 µM	An anticancer and antiviral agent with multiple mechanisms of action	164,3864595	53,90292388
